# Reconstructing complex lineage trees from scRNA-seq data using MERLoT

**DOI:** 10.1093/nar/gkz706

**Published:** 2019-08-20

**Authors:** R Gonzalo Parra, Nikolaos Papadopoulos, Laura Ahumada-Arranz, Jakob El Kholtei, Noah Mottelson, Yehor Horokhovsky, Barbara Treutlein, Johannes Soeding

**Affiliations:** 1 Quantitative and Computational Biology Group, Max Planck Institute for Biophysical Chemistry, Am Fassberg 11, 37077 Goettingen, Germany; 2 Genome Biology Unit, European Molecular Biology Laboratory, Meyerhofstraße 1, 69117 Heidelberg, Germany; 3 Department of Evolutionary Genetics, Max Planck Institute for Evolutionary Anthropology, Deutscher Platz 6, 04103 Leipzig, Germany; 4 Department of Biosystems Science and Engineering, ETH Zürich, Basel, Switzerland

## Abstract

Advances in single-cell transcriptomics techniques are revolutionizing studies of cellular differentiation and heterogeneity. It has become possible to track the trajectory of thousands of genes across the cellular lineage trees that represent the temporal emergence of cell types during dynamic processes. However, reconstruction of cellular lineage trees with more than a few cell fates has proved challenging. We present MERLoT (https://github.com/soedinglab/merlot), a flexible and user-friendly tool to reconstruct complex lineage trees from single-cell transcriptomics data. It can impute temporal gene expression profiles along the reconstructed tree. We show MERLoT’s capabilities on various real cases and hundreds of simulated datasets.

## INTRODUCTION

### Background

Recent advances in single-cell sequencing techniques (1–3) permit to measure the expression profiles of tens of thousands of cells making ambitious projects like the single-cell transcriptional profiling of a whole organism (4) or the Human Cell Atlas (5) possible. These efforts will better characterize the different cell types in multicellular organisms and their lineage relationships (6). The advances also put within reach the question of how single cells develop into tissues, organs or entire organisms, one of the most fascinating and ambitious goals in biology that would also have wide-ranging consequences for the study of many human diseases.

It is critical to develop methods that can reliably reconstruct cellular lineage trees that reflect the process by which mature cell types differentiate from progenitor cells. This is challenging due to the inherently high statistical noise levels in single cell transcriptomes, the high-dimensionality of gene expression space and the strong non-linearities among gene interactions due to multiple transcriptional programs running in parallel for specifying the different cell type identities (6).

Different methods have been developed in the last years for inferring single-cell trajectories (7,8). Most of these methods first apply a manifold embedding in order to reduce the dimensionality of the problem and then implement various strategies for reconstructing the trajectory structure on it. Some tools are intended for linear topologies, while others aim to resolve bifurcations, multifurcations or even complex trees with many internal branchpoints. The latter case has proven very challenging, and there is much room for improvement. Here we present MERLoT (MEthod for Reconstructing Lineage tree Topologies), a tool that can reconstruct highly complex tree topologies containing multiple cell types and bifurcations.

MERLoT uses a low-dimensional embedding to reconstruct the cellular lineage tree topology and then maps this topology to the original high-dimensional expression space. Different manifolds have been shown to be useful for the reconstruction of different lineage trees. MERLoT implements diffusion maps (9) as produced by the Destiny package (10) as the default method for dimensionality reduction. However, users can provide MERLoT with any low-dimensional space coordinates to perform the tree reconstruction.

MERLoT explicitly models the tree structure, defining its endpoints, branchpoints and locating a set of support nodes between these that act as local neighborhoods for cells. This model-based strategy gives insights into the temporal order of branching and the emergence of intermediary cell types. Once the lineage tree has been reconstructed in the low dimensional space, MERLoT is able to embed it back to the high dimensional gene expression space. The support nodes play a 2-fold role in this step: they integrate the gene expression information of the cells assigned to them, and they inform the gene expression profiles of nearby support nodes. This reduces the overall noise levels, interpolates gene expression values for lowly sampled regions of the lineage tree and imputes missing expression values.

We show MERLoT’s performance on several real datasets, using different manifold embeddings and on hundreds of simulated datasets. We generated a total of 2000 synthetic datasets with PROSSTT (11), divided into subsets of 100 simulations containing from 1 to 10 bifurcations. To the best of our knowledge, this benchmark datasets is the largest and most complete one up to date (available at http://wwwuser.gwdg.de/∼compbiol/merlot/). We show that MERLoT outperforms other methods by producing a better classification of cells to the different branches that constitute the lineage trees. This is crucial when studying the progression of gene expression along the different trajectories in the tree, since a sub-optimal classification of cells mixing different cell types together leads to inaccurate imputation of gene expression time courses and impairs downstream analysis (3).

We repeated the benchmark with simulations generated by another tool, Splatter (12). For more information, details about method performance, and divergence analysis of the simulations, please refer to the Supplementary Note 3.

MERLoT is implemented as an R package and publicly available at https://github.com/soedinglab/merlot. MERLoT allows users to easily retrieve subpopulations of cells that belong to specific branches or belong to specific paths along the tree. It can also calculate pseudotime assignments, impute pseudotemporal gene expression profiles or find genes that are differentially expressed on different tree segments.

## MATERIALS AND METHODS

### MERLoT’s workflow and section summary

Given an expression matrix with *N* cells as rows and *G* genes as columns, a manifold embedding technique can project the data onto a number of informative dimensions *D* <<*G*. Since many dimensionality reduction techniques project the data onto mutually orthogonal dimensions, two or three dimensions often do not capture the true topology of the data. In practice, we have found that for topologies with *N* branches we needed *N* + 1 dimensions for optimal results. Determining the correct number of dimensions to use is not trivial, and using more dimensions than needed might introduce undesired noise.

The rest of the ‘Materials and Methods’ section is structured to explain the different steps that are followed by MERLoT after dimensionality reduction for lineage tree reconstructions and downstream analysis that are further explained in the following subsections:

(i) Scaffold Tree Reconstruction.
(ii) Elastic Principal Tree (EPT) calculation in low dimensional space.
(ii) EPT Embedding into the gene expression space.
(iv) Pseudotime assignment.
(v) Differentially expressed genes detection.

Additionally to the aforementioned features, MERLoT offers several functions to allow users to perform further analysis, exemplified by a correlation network reconstruction using MERLoT’s gene imputed values explained in the section:

(vi) Correlation Network Construction:

We describe the datasets used in the manuscript as well as the benchmark we performed to compare to other tools using synthetic data in the sections:

(vii) Real Datasets
(viii) Benchmark on Synthetic Datasets

More detailed descriptions of different algorithmic steps can be found in Supplementary Note 1, and an overview of MERLoT in pseudocode form can be found in Supplementary Note 2.

#### Terminology

We model cellular lineage trees such as the ones that result from single-cell snapshots of differentiating populations with trees as defined in graph theory, i.e. undirected graphs in which any two vertices are connected by exactly one path (13). In the context of an EPT, each node is referred as a support node in the lower dimensional space. When embedding the EPT into the gene expression space $\mathbb {R}^G$, support node v_*n*_ is referred to as *pseudocell n*, since it contains the imputed gene expression values based on the cells assigned to them.

For illustration purposes, consider an experiment where quiescent progenitor cells *A* are given a differentiation signal, mature for a time period and then either differentiate to specific progenitors *B* or become fully differentiated cells *C*. Nodes with exactly one neighbor are called *endpoints*. They correspond to ending or starting points of the process captured in the experiment (*A, B* and *C*). Nodes with more than two neighbors are named *branchpoints*. In this example it will be the node where the maturation ends and the cell fate decision is made. Paths between endpoints and branchpoints or between two branchpoints are named *branches*. The collection of endpoints, branchpoints and their connectivity is the *topology* of the tree. For example, a tree (*BC, BD*)*AB*; (Newick format; for more information see http://evolution.genetics.washington.edu/phylip/newick_doc.html) describes a tree with a single bifurcation. It has three endpoints *A, C, D*, one branchpoint *B* and three branches, *AB, BC* and *BD*. We refer to trees with many branchpoints as having *complex* topologies.

### Scaffold tree reconstruction (Figure 1B)

We calculate the shortest paths *p*_*ij*_ between all pairs of cells *i* and *j* that minimize the squared Euclidean distance $d_{ij}^{2}$ (using the distance *d*_*ij*_ would only discover the edge *i, j* and not a longer path). We use a modified version of the csgraph module from the scipy (https://www.scipy.org/) library, available at https://github.com/soedinglab/csgraph_mod.

The shortest path *p*_*kl*_ that maximizes the number of cells *S*_*kl*_ (or the longest total euclidean distance in case of ties) is added to the tree *T* and the cells *k, l* added to the set of endpoints of the lineage tree $\mathcal {E}$ (Supplementary Figure S1). Additional endpoints *n* are iteratively added to the tree by selecting the shortest paths *p*_*ni*_, *i* ∈ *T* that maximize $s_\mathcal {E}(n)$, the number of cells added to *T*:

(1) $$\begin{equation*} s_\mathcal {E}(n) := 0.5 \times \min \lbrace S_{kn} + S_{nl} - S_{kl} \, :\, k,l \in \mathcal {E} \rbrace . \end{equation*}$$

**Figure 1.**
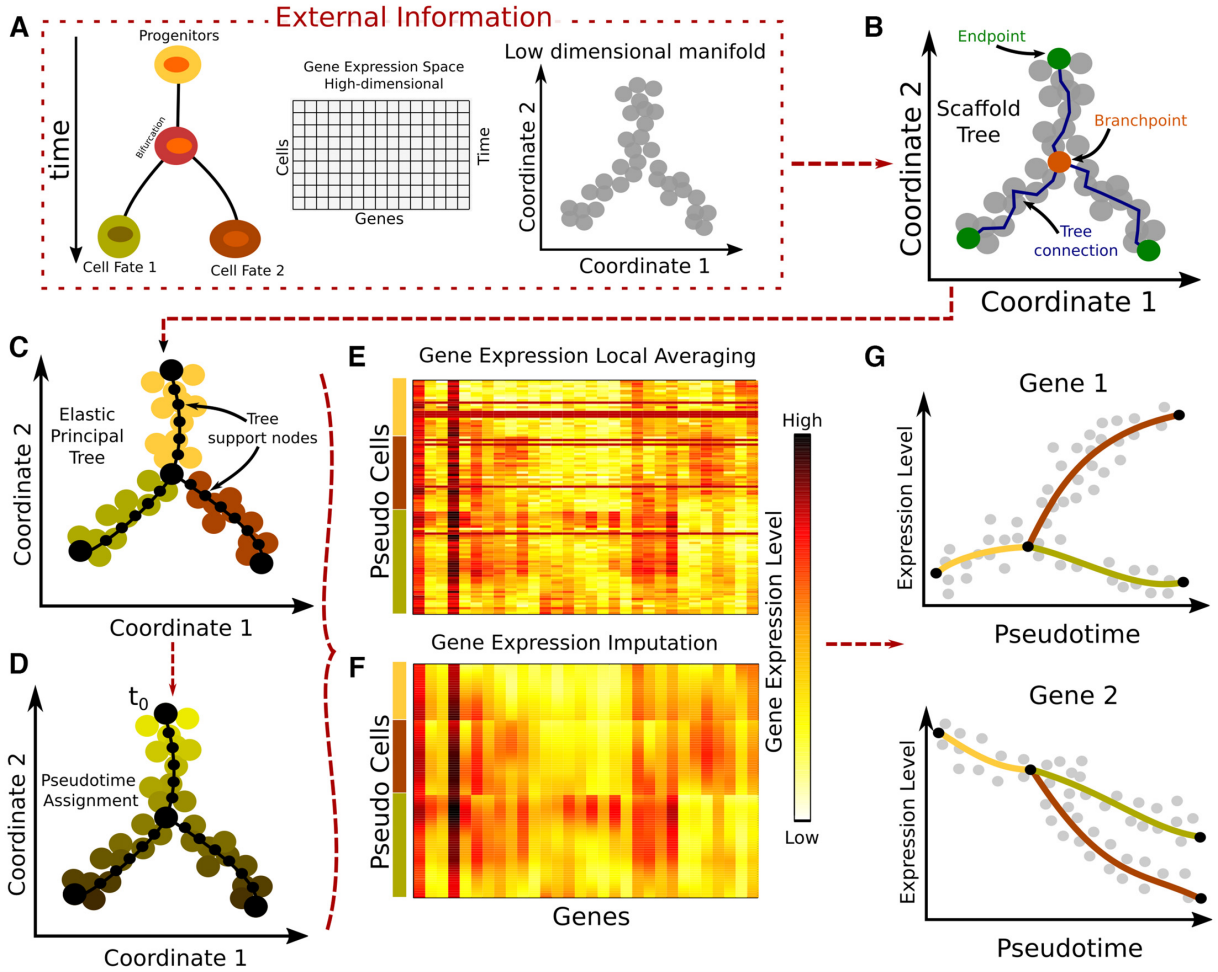
MERLoT’s workflow: (**A**) Input to MERLoT is a gene expression matrix sampled from a dynamic process in which several cell types are present. MERLoT uses diffusion maps to reduce the dimensionality of the expression vectors for each cell to a few components (typically between 2 and 20). Users can provide any low-dimensional manifold set of coordinates to MERLoT as input. (**B**) A scaffold tree is calculated given the low-dimensional manifold coordinates. (**C**) The scaffold tree is used to initialize a principal elastic tree, composed of *k* support nodes (default: 100), on which cells are assigned to the different branches of the tree. (**D**) Given a cell or tree node as the initial pseudotime t_0_, pseudotime values propagate to the rest of cells/support nodes proportional to the distance along the tree that separates them from t_0_. (**E**) Expression values from cells assigned to a given support node or pseudocells (see main text) are averaged to provide the expression profile of each gene. (**F**) Gene expression values after imputation and interpolation in the gene expression space: A high-dimensional principal elastic tree is initialized with the connectivity from the low dimensional principal elastic tree plus the averaged expression values from the support nodes to impute smoothed gene gene expression data for each gene in the gene expression space. (**G**) MERLoT imputes the pseudotime-dependent expression profile of each gene along each branch in the tree. Gene expression can be visualized as a function of pseudotime.

In ‘auto’ mode every time a new endpoint is proposed we evaluate if $\max \lbrace s_\mathcal {E}(n^{\prime }) : 1 \le n^{\prime } \le N, n^{\prime } \notin \mathcal {E} \rbrace >\sqrt{N}$ holds true. Otherwise, we calculate the branchpoints and tree connectivity for the endpoints in $\mathcal {E}$, including *n*, using the methodology explained in the next subsection. After this, all cells are mapped to their closest branch. If the branch added by the selected *n* endpoint contains more than MinBranchCells$=\sqrt{N}$ cells mapped to it, the branch is kept in the tree scaffold structure and the endpoints search is repeated. Otherwise, the endpoint search terminates and *n* is discarded as endpoint. The MinBranchCells threshold can be modified by the user. Alternatively, instead of using a stop criterion, users can set the number of endpoints that are aimed to be found (fixed mode) regardless of the branch lengths.

After locating all endpoints we use the Neighbor Joining (NJ) criterion (14) in order to derive a tree (Supplementary Figure S2). Let $\mathcal {V}$ be the set of yet unprocessed endpoint and branchpoint nodes of the tree. We initialize $\mathcal {V} \leftarrow \mathcal {E}$ with the endpoint set $\mathcal {E}$.

We pick the two nodes *k, l* in $\mathcal {V}$ that are guaranteed to be next neighbors and therefore can be linked via a single branchpoint by minimizing the NJ distance $d^\mathrm{NJ}_{kl}$:

(2) $$\begin{equation*} d^\mathrm{NJ}_{kl} := S_{kl} - \frac{1}{|\mathcal {V}-2|} \sum _{m \in \mathcal {V}} (S_{mk} + S_{ml}) \, . \end{equation*}$$

The branchpoint cell *m* between *k, l* has a minimal distance from *k* and *l* as well minimal average distance from all other nodes in $\mathcal {V}$:

(3) $$\begin{equation*} m = \arg _m \min \left\lbrace S_{km} + S_{lm} + \frac{\sum \limits _{n \in \mathcal {V} \backslash \lbrace k, l\rbrace }{S_{nm}}}{|\mathcal {V}-2|} \right\rbrace \, . \end{equation*}$$

In $\mathcal {V}$*k* and *l* are replaced by *m*, while the edges *l* − *m* and *m* − *k* are added to the tree (Supplementary Figure S2). This is repeated until $|\mathcal {V}| = 2$, where the remaining nodes trivially fulfill the criterion and can be joined. Note that the same cell can be detected more than once as a branchpoint.

#### Local averaging mode

Dijkstra’s shortest path algorithm has, in the scipy implementation that MERLoT uses, a time complexity of $\mathcal {O}(Nk + N\mathrm{log}(N))$ where *N* is the number of nodes (cells) and *k* the average number of connected edges per node (cell). During the scaffold tree calculation, MERLoT calls Dijkstra’s algorithm for every cell, leading to an overall complexity of $\mathcal {O}(N(Nk + N\mathrm{log}(N)))$. Since MERLoT does not impose a cut-off on *k*, it is equal to *N*, and the complexity becomes $\mathcal {O}(N^3 + N^2\mathrm{log}(N))$. This means that a linear increase in cell number leads to more than a cubic increase in complexity.

To speed up the calculation of the scaffold tree, we implemented a local averaging strategy. Given a number of centroids we cluster the manifold coordinates of the cells with k-nearest neighbors (knn) (15) and subsequently calculate the scaffold tree on the cluster centroids. This knn-reduced scaffold tree is then used as input to the EPT, which returns a knn-reduced elastic tree. This can then be inflated with the original manifold coordinates. For the ‘deep’ benchmark (see Section) we reduced coordinates to $4 \sqrt{N}$ cells, for an effective complexity of $\mathcal {O}(64N\sqrt{N} + 16N\mathrm{log}(4N))$.

Apart from a massive speed-up, the local averaging strategy also made the quality of the elastic trees less dependent on the choice of elasticity hyperparameters (see next subsection). Local averaging adds little value for small datasets, as averaging over very limited samples only worsens the signal-to-noise ratio. We recommend that MERLoT should be used with local averaging for large datasets (more than a few thousand cells; also see Supplementary Figure S7).

### Elastic principal tree in the low dimensional manifold (Figure 1C)

To produce smoother, more homogeneously interpolated lineage trees MERLoT uses the EPT algorithm (16,17) as implemented in the ElPiGraph.R module (https://github.com/Albluca/ElPiGraph.R and described on arXiv: https://arxiv.org/abs/1804.07580). The EPT algorithm is used to approximate the distribution of cells in a given space with a tree structure composed of *k* nodes. Direct application of the EPT algorithm is unstable as it often returns trees that are manifestly far from the global optimum (e.g. wrong number of endpoints or grossly misplaced branchpoints). This can be observed in the tree reconstructions that were performed using the non-initialized EPT using ElPiGraph (Supplementary Figure S19). The recovered tree topologies have more (small) branches than MERLoT reconstructions, while neighboring bifurcations found by our method get collapsed. We use the computeElasticPrincipalCurve function from ElPiGraph, that we initialize with the coordinates of the scaffold tree endpoints and branchpoints and the edges among them. This function will not change the scaffold tree topology used as an initialization point but add nodes to the EPT by iterative bisection of edges until it reaches the specified number of *k* support nodes. As a result a smoothed version of the scaffold tree is obtained.

**Figure 2.**
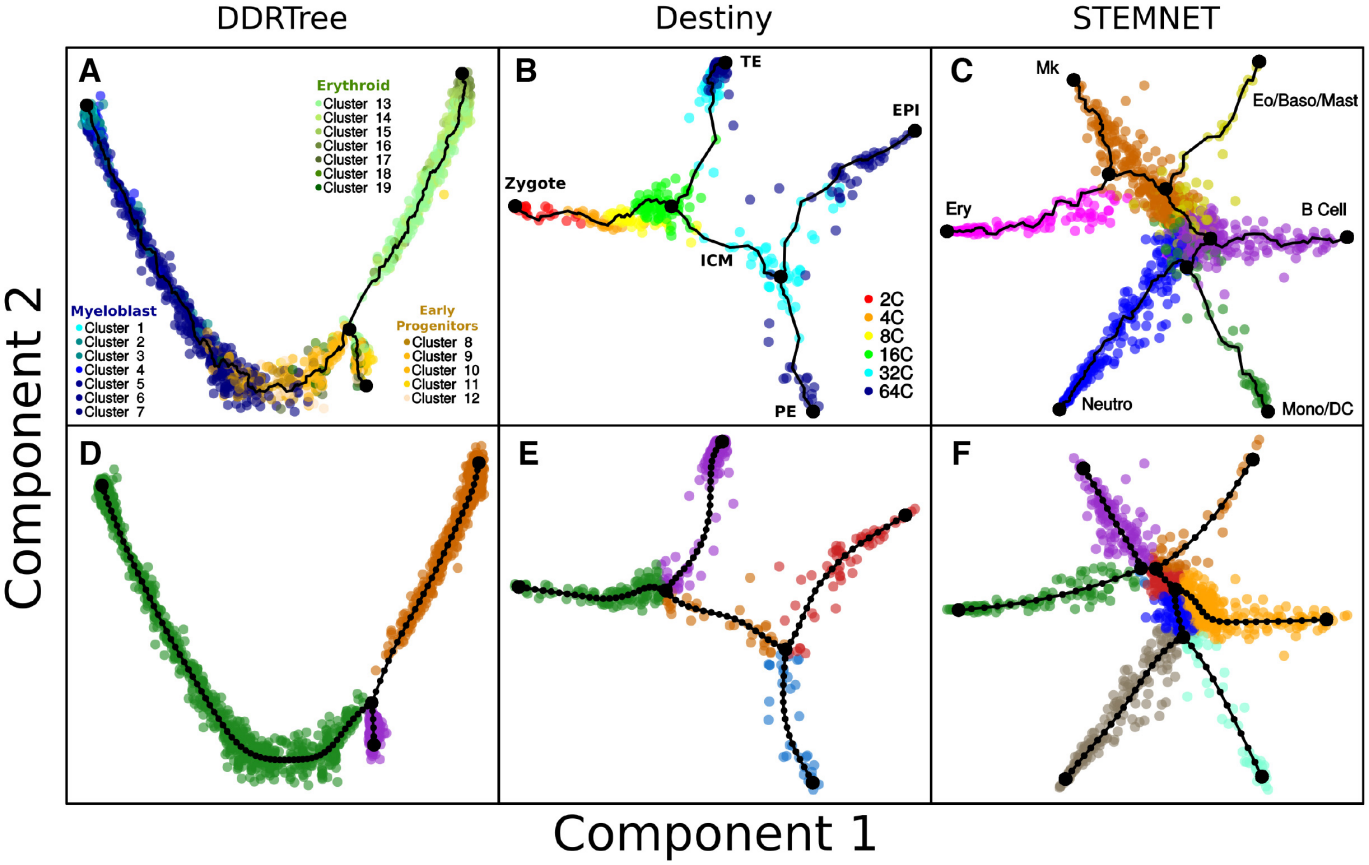
MERLoT’s scaffold tree reconstructions: in combination with (**A**) DDRTree coordinates for analyzing the data from Paul *et al.* (22); (**B**) diffusion maps coordinates for analyzing the data from Guo *et al.* (23) (diffusion components 2 and 3 rotated around component 1 for better visualization of the data); (**C**) STEMNET coordinates for analyzing the data from Velten *et al.* (24). Cells are colored according to cell type annotations provided by the authors of each dataset. (**D–F**) EPT reconstructions using the scaffold trees from panels A–C, respectively, as an initialization point. Cells are colored according to MERLoT’s branch assignments.

We performed a grid search around the default EPT hyperparameter values by visually examining reconstructed EPTs with *k* = 100 support nodes on the datasets shown in Figure 2. We obtained μ_0_ = 0.0025 and λ_0_ = 0.8 · 10^−9^, values that have held up well for simulated datasets (see below). For different values of *k* we adjust according to μ = (*k* − 1)μ_0_ and λ = (*k* − 2)^3^λ_0_. All reconstructions in our benchmark were performed with the standard function using *k* = 100.

For some particular topologies μ and λ might need to be tuned in order to produce optimal results, in particular if *k* is increased a lot. Alternatively, MERLoT can bisect the edges in a given EPT, by additional nodes producing a new EPT with almost 2*k* support nodes. Note that these are special cases. For future development of MERLoT we plan to introduce a fitness function to automatically optimize the hyperparameters individually on each dataset by maximizing the log likelihood of the EPT. An in-depth discussion of elasticity hyperparameters can be found in the Supplementary Material.

During the revision of this article a new tool called STREAM has been published that also exploits our strategy of using EPTs (18). STREAM exploits EPTs to find the tree structure in the embedded low dimensional space (Modified Locally Linear Embedding, MLLE). As mentioned, MERLoT can reconstruct trees in any low dimensional representation. Additionally, MERLoT exploits the EPT algorithm to embed the low-dimensional tree structure into the high-dimensional gene expression space and hence obtains imputed gene expression values (see next subsection).

### Elastic principal tree embedding into the gene expression space (Figure 1E and F)

First, cells are assigned to their closest support node according to euclidean distance in manifold space. Their average expression profile is used to initialize the ‘expression profile’ of the node to which we will refer now as a ‘pseudocell’. Nodes without cells assigned to them are initialized with a null vector. By constructing such ‘pseudocells’, we translate the positions of the support nodes in the low-dimensional manifold space to approximate positions in the full gene expression space.

Finally, the EPT algorithm is initialized with the average expression profiles of the pseudocells and a list of edges representing their connectivity in the low dimensional EPT to calculate an EPT in the high-dimensional gene expression space. As a result the pseudocell profiles are updated with imputed values based on the cells from which the initial averaged values were calculated.

### Pseudotime assignment (Figure 1D)

Pseudotime is a quantitative measure of the progress of a cell through a biological process (19). Given the reconstruction of a lineage tree by MERLoT, cells can be assigned pseudotime values as a function of the number of edges along the structure that separate them from the initial point of the process. MERLoT automatically sets the initial pseudotime, *t*_0_, to one of the first two detected endpoints. Users can also set *t*_0_ to any endpoint, branchpoint or to any individual cell. In the latter case, the closest node to that cell will be assigned as *t*_0_ and the pseudotime values for the other nodes will be assigned as before. After a pseudotime value is assigned to each support node, cells will take the pseudotime value from their closest node in the tree. Alternatively, cells can be projected to the edge that connects their two nearest support nodes and thereby receive continuous pseudotime labels. MERLoT can calculate pseudotime in both the low-dimensional manifold space and in the high-dimensional gene expression space.

### Differentially expressed genes detection

**Figure 3.**
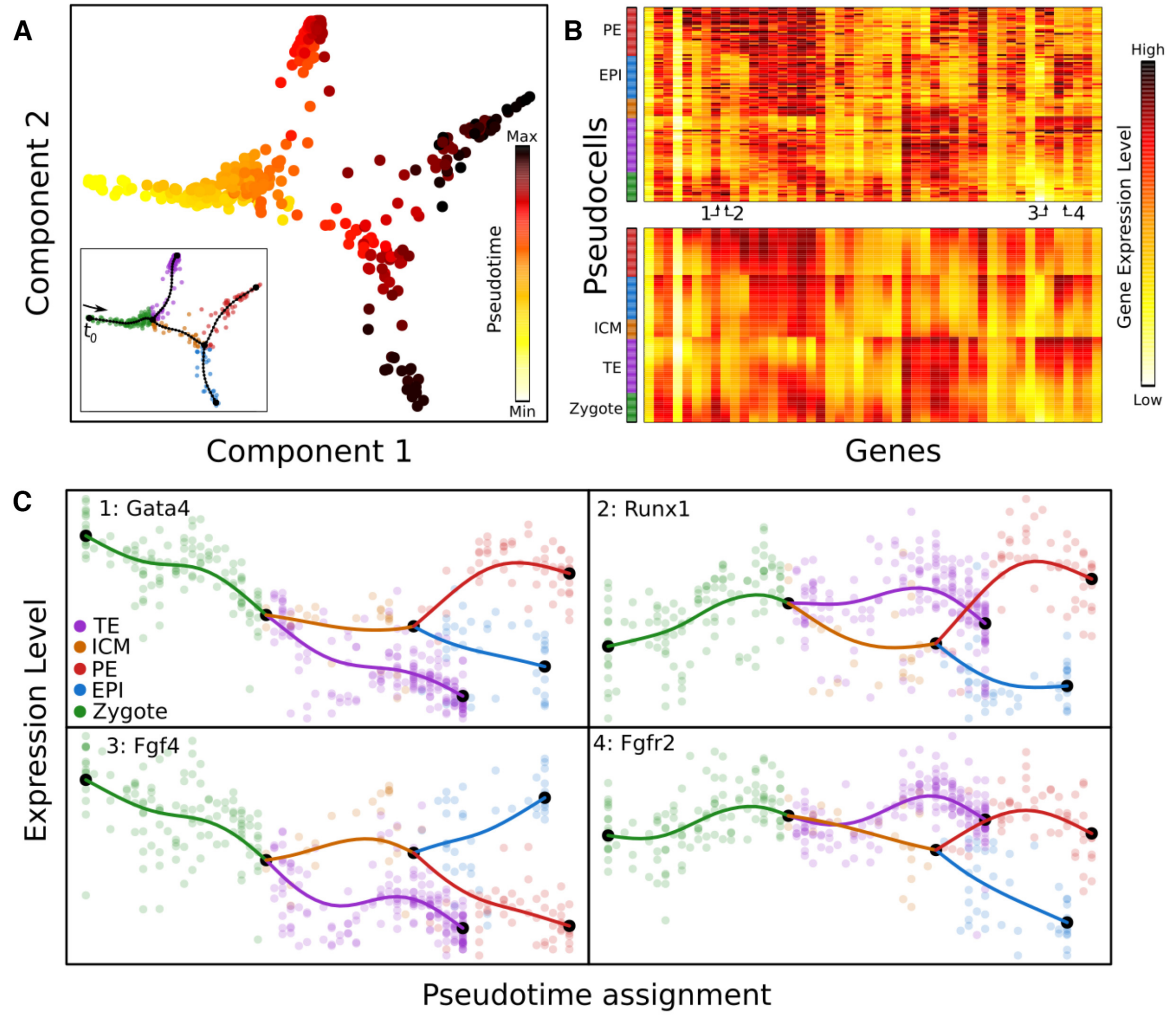
Pseudotime assignment and interpolation of gene expression profiles: (**A**) Pseudotime assignment in color code for cells from Figure 2B and E, taking the zygote state as *t*_0_. (**B**) Color-coded matrix of gene expression values for tree pseudo cells (rows) times genes (columns) before (top) and after (bottom) the gene expression space interpolation using the EPT. Pseudo cells are ordered according to their pseudotime and genes were hierarchically clustered. Numbers indicate specific genes shown in panel C. The color code in the bar on the left side of the heatmaps refers to pseudo cells branch assignments. (**C**) Gene expression profiles over pseudotime for four genes that are differentially expressed between the EPI and PE lineages. Semi-transparent circles represent the expression values of individual cells and solid lines correspond to MERLoT’s interpolations. Colors as in Figure 2E.

After a linear tree reconstruction has been performed, MERLoT can easily find groups of genes being differentially expressed among different groups of cells. If two groups of cells are provided, e.g cells assigned to two branches in the tree (Figure 3C), MERLoT performs a Kruskal–Wallis rank sum test (see Supplementary Note 4 for details) to evaluate which genes in the full expression matrix are differentially expressed on them. If a single subpopulation of cells is provided, the comparison is made against the rest of cells in the data. The entire list of genes is given as output, ordered by the test *P*-values results. Also, *e*-values are provided by multiplying the *P*-values by the number of *G* genes being tested.

### Correlation network reconstruction (Figure 8)

We performed a Gene Correlation Network (GCN) reconstruction for the fibroblasts to neurons transdifferentiation dataset from the Treutlein group (20). We reconstructed the lineage tree, reconstructed the GCN, performed gene clustering and differential gene expression analysis. The script for performing this analysis is available at https://github.com/soedinglab/merlot/tree/master/inst/example/.

#### Network construction

Given the expression profile of a gene in all cells (non-imputed values) or in the tree support nodes (imputed values) we create a matrix that contains the pairwise Pearson’s correlation coefficient between all pairs of genes. Given this matrix we can use the R package *igraph* (http://igraph.org/r/) by defining a threshold to decide which Pearson’s correlation coefficients to include as weights for the edges in the graph. For the correlation measures and cut-offs used, please refer to Supplementary Figures S10–14.

#### Cluster analysis

We clustered the genes on the GCN by applying the *walktrap* algorithm as implemented in the *igraph* package. This algorithm partitions a graph into densely connected parts of a network (modules) by exploiting the fact that short random walks tend to stay within a module (21). Any module with less than a specified lower boundary of nodes is dissolved and those genes are considered as unclustered. We set the minimum number of genes to 3.

#### Gene ontology term enrichment analysis

To help determine the function of network defined clusters, a module for Gene Ontology (GO) term enrichment was implemented. For this purpose the R package topGO was used. Enrichment was computed by the Fisher exact test with the gene set of the data set as the gene universe. Gene ontology tables per cluster were retrieved and representative keywords were selected by cluster.

#### Differentially expressed genes

We detected the differentially expressed genes at every branch of the reconstructed lineage tree using the branch_differential_expression function from MERLoT. All genes are ranked according to the *e*-value obtained from the Kruskal–Wallis rank sum test, applied to test whether they are differentially expressed or not. Genes with *e*-value < 10^−3^ are considered to be differentially expressed. Genes are colored according to the mean difference in expression between the two compared sets of cells (i.e. selected branch and rest of the tree for each case). Upregulated genes are shown in shades of red and downregulated genes in shades of blue. Intensity corresponds to log fold change of gene expression. Genes that are not significantly differentially expressed are colored in black.

### Real datasets

#### Myeloid progenitors differentiation (Paul *et al.*, (22))

This dataset was produced applying massively parallel single-cell RNA-seq (MARS-seq) which uses unique molecular identifiers (UMIs). After quality control and selection of informative genes the expression matrix contains 2730 cells and 3459 genes. Originally the authors reported 3461 informative genes with some of them being incorrectly formatted as dates, e.g. 5-Mar, 4-Sep. We were able to correct the IDs of most of them to valid GeneIDs except two (IDs: 7-Sep and 2-Mar) which were excluded from the analysis (https://github.com/soedinglab/merlot/tree/master/inst/example/ExamplePaul2015.R).

#### Mouse zygote to blastocyst (Guo et. al, (23))

The dataset was produced by the Biomark RT-qPCR system and contains Ct values for 48 genes measured in 442 mouse embryonic stem cells at seven different developmental time points, from the zygote to blastocyst (23). The data was cleaned and normalized by following the vignette from the Destiny package. A total number of 428 cells and 48 genes were kept in the final expression matrix. A diffusion map was calculated using Destiny and the first three diffusion coordinates were used to calculate the lineage tree. We rotated the cells and tree nodes coordinates around the first axis in order to produce a two-dimensional representation of the data and improve visualization (see Figure 2B, E and https://github.com/soedinglab/merlot/tree/master/inst/example/ExampleGuo2010.R).

#### Haematopoietic stem and progenitor cells (HSPCs) (Velten *et al.*, (24))

The scRNA-seq data was generated samples taken from two donor individuals (smart-seq2.HSC for individual 1 and QUARTZ-seq for individual 2), with all findings systematically compared between them. We followed the vignette for the STEMNET software available as part of its R package and obtained the normalized data, the cell types labels, and the STEMNET coordinates (see Figure 2C, F and https://github.com/soedinglab/merlot/tree/master/inst/example/ExampleVelten2017.R).

### Benchmark on synthetic datasets

We evaluated the performance of MERLoT and other lineage tree reconstruction methods on synthetic data. We produced two simulation sets with PROSSTT (11) and one more with Splatter (12). Each set contains 1000 (10 × 100) datasets with 1–10 bifurcations (3–12 endpoints) each. The topology of each dataset was created by successively adding a bifurcation to a random end point until the desired number of bifurcations was reached (Supplementary Figure S8). In the first PROSSTT set (‘lean’) we sampled for each lineage tree 50 cells from every branch, while in the second (‘deep’) we used the same lineage trees but sampled 500 cells from each branch. In the Splatter set we sampled 100 cells from each branch.

PROSSTT generates a simulated scRNA-seq dataset in four steps: (i) it generates a tree (number and length of branches, connectivity), (ii) it simulates average gene expression levels μ_*g*_(*t, b*) (pseudotime *t*, branch *b*), (iii) it samples points in the tree (*t, b*) (4) it retrieves μ_*g*_(*t, b*) for each sampled point and draws UMI counts from a negative binomial distribution.

We provide the scripts used to create the simulations (https://github.com/soedinglab/merlot-scripts) as well as the simulations themselves (http://wwwuser.gwdg.de/∼compbiol/merlot/). Detailed information about the simulation procedure and parameter values can be found in the Supplementary Material.

Splatter takes a slightly different approach to the simulation of lineage trees (‘paths’ in the terminology of Splatter). It is a software primarily designed to simulate populations of cells with differential expression between them. In order to simulate a lineage tree, it simulates populations with differential expression at each successive waypoint of the lineage tree (between start and first branchpoint, between successive branchpoints, between branchpoints and endpoints), and then simulates how gene expression changes from one waypoint to the other.

For parameter selection, we kept default parameters as far as the count model and the generation of average gene expression values were concerned (parameters controlling mean, library size, expression outlier, biological coefficient of variation). The rest of the simulation parameters were picked to mirror those in the PROSSTT simulations (see Supplementary Material).

We visualized typical prediction examples for three topologies and several methods in Supplementary Figure S5.

## RESULTS AND DISCUSSION

### MERLoT’s workflow for lineage trees reconstruction and gene expression imputation

Given the matrix of expression values for all cells (Figure 1A), MERLoT reconstructs lineage trees according to the following steps (for details see ‘Materials and Methods’ section): First, MERLoT applies a dimensionality reduction method to map the high-dimensional expression vectors of cells to a low-dimensional space. Users can replace the default, diffusion maps, with the method of their choice. Second, MERLoT calculates a scaffold tree in the low-dimensional space combining the Dijkstra’s shortest path (25) and NJ (14) algorithms to define the location of endpoints, branchpoints and their connectivity (Figure 1B). (View Supplementary Note 5 for a pseudocode explanation). The scaffold tree is used as initialization for calculating an EPT (16) (see Methods for details). The EPT smooths the scaffold via an optimization procedure that places a user-defined number of support nodes between endpoints and their corresponding branchpoints interpolating the density of cells in the low-dimensional space (Figure 1C). Once the low-dimensional tree is optimized, an initial pseudotime t_0_ is assigned to the user-specified tree root. The pseudotime of each cell is then proportional to its distance from the root along the tree structure (Figure 1D).

To study gene expression changes along the different tree branches, MERLoT embeds the low-dimensional EPT structure into the high-dimensional gene expression space. Each tree support node in the low-dimensional space is mapped one-to-one to a tree support node in the gene expression space: we first assign each cell in the low-dimensional space to its nearest support node. Then, we initialize the corresponding support node in the gene expression space to the average gene expression level of all cells assigned to it (Figure 1E), and we run the EPT algorithm again (‘Materials and Methods’ section). In this way, we find the gene expression values of all the support nodes (Figure 1F), which can be considered as ‘pseudocells’, representing waypoints in the idealized cell differentiation paths and containing imputed gene expression values for their surroundings in the expression space.

The cells’ pseudotime values can also be refined in this step, since cells are reassigned to support nodes in the full multi-dimensional space. By combining the imputed expression values with the pseudotime assignments of the support nodes, MERLoT can reconstruct imputed pseudotime courses of gene expression profiles along the tree (Figure 1G).

### Applying MERLoT to real datasets

We applied MERLoT on three real datasets with different degrees of lineage tree structure complexity (details in ‘Materials and Methods’ section): (i) scRNA-seq data (with Unique Molecular Identifiers, UMIs) for myeloid progenitor differentiation (2730 cells, 3460 genes) (22), embedded in DDRTree coordinates (Figure 2A and D), (ii) single-cell quantitative polymerase chain reaction (qPCR) data for zygote to blastocyst differentiation (428 cells, 48 genes) (23), embedded in a diffusion map (Figure 2B and E) and (iii) scRNA-seq data (index-omics) for haematopoietic stem and progenitor cells (1034 cells, 469 genes), using STEMNET coordinates (24). The number of endpoints of the lineage trees, found by MERLoT in ‘auto’ mode, are consistent with the expected number of cell types that are described to be present on each of the analyzed datasets.

After the lineage tree reconstruction, each support node on the tree will be assigned a pseudotime value equal to the number of edges that separate it from the beginning of the differentiation, *t*_0_. This can be a tree endpoint (the Zygote branch in Figure 2B) or an internal support node (for example a node in the red branch of Figure 2E). Each cell will be assigned the pseudotime value of its closest tree node. In Figure 3A, pseudotime values for the zygote to blastocyst dataset are shown. Because scRNA-seq data contain a lot of technical and biological noise (6), cells with similar pseudotime values may have large variations in gene expression. MERLoT imputes denoised gene expression profiles by embedding the reconstructed lineage tree into the original gene expression space.

This model-based interpolation results in denoised pseudotime courses of gene expression for the entire tree. As an example, Figure 3C shows the expression profiles of four genes that are differentially expressed between the epiblast (EPI, in red) and the primitive endoderm (PE, in blue) cell lineages (Methods). Note how the expression values of the pseudocells (solid lines) interpolate and smooth the noisy single-cell expression values (circles) even in regions with low cell density.

### Lineage tree reconstruction in high-dimensional space

While two or three-dimensional projections of datasets are easier to visualize, MERLoT can utilize any number of informative dimensions of the embedding space to reconstruct a lineage tree. However, visualizing hundreds or thousands of cells in multiple dimensions is challenging. MERLoT overcomes this limitation by using graph drawing techniques to project complex multidimensional EPTs into two dimensions while still displaying cell annotation and density. In Supplementary Figure S3 we show reconstructed topologies for simulations with 6, 8, 10 and 12 different cell types.

**Figure 4.**
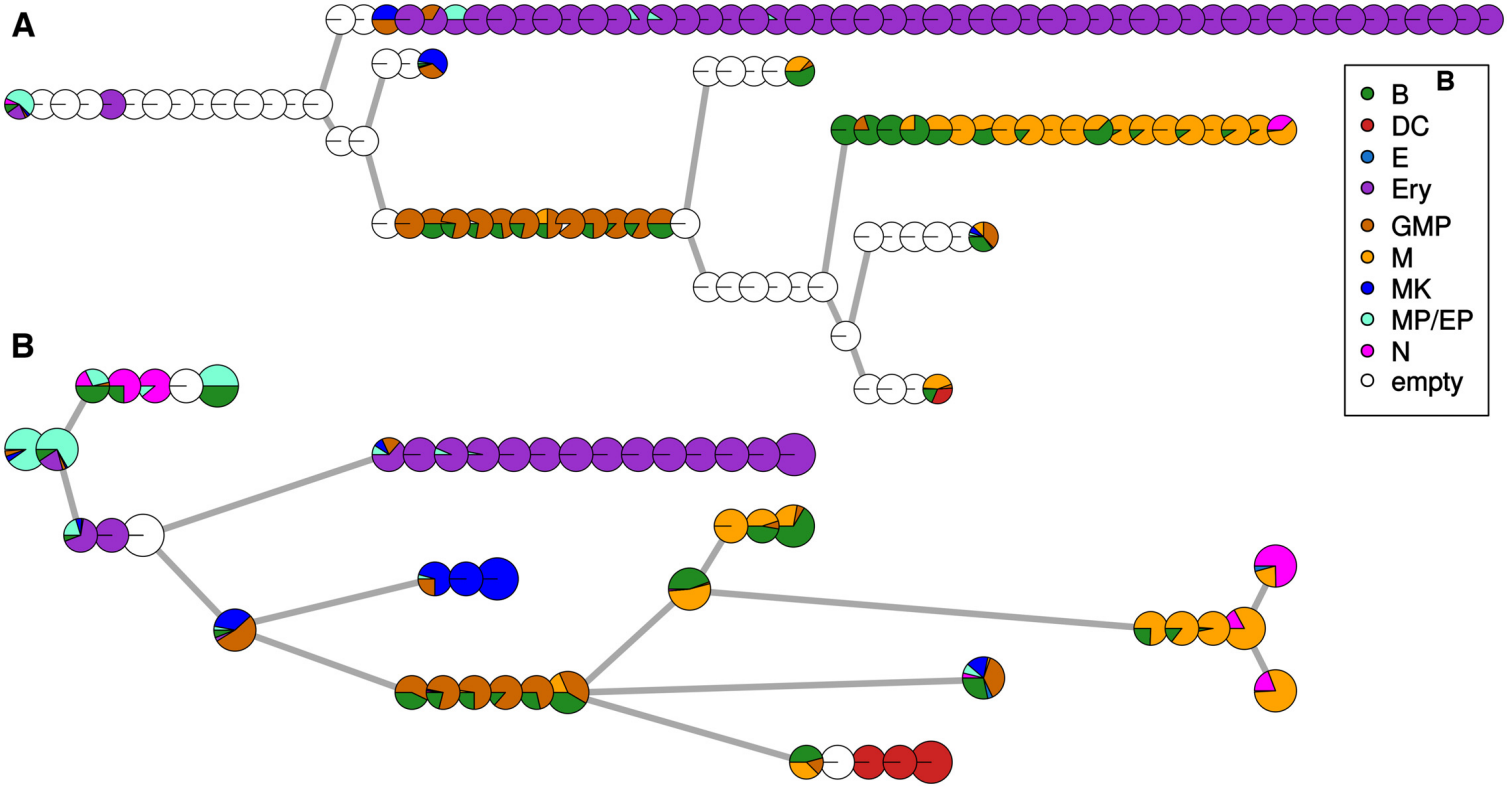
Reconstruction of haematopoietic system B: basophil (green); DC: dendritic cell (red); E: eosinophil (light blue); Ery: erythrocyte (purple); GMP: granulocyte and monocyte progenitor (brown); M: monocyte; MK: megakaryocyte; MP/EP: multipotent myeloid and erythroid progenitors; N: neutrophil (orange). Each pie chart represents a tree node, and the colors denote the different cell types mapped to it. (**A**) The Monocle2 reconstruction of the process. Each pie chart is one of the nodes of the minimum spanning tree. (**B**) The MERLoT reconstruction of the process. Each pie chart is a node of the EPT. Pie charts corresponding to branchpoints and endpoints are larger just for aesthetic purposes

In the original Monocle2 paper (26), the authors show in Supplementary Figure S16 their analysis of the haematopoiesis dataset produced by Paul *et al.* (22). They use 10 components of the DDRTree projection to recover a topology with five branching points. We applied MERLoT on these coordinates and visualized the resulting tree using as annotation the cell types that Paul *et al.* assigned to the various clusters (Figure 4; also see Figure Supplementary Note 6 in the Supplementary Material).

Monocle2 (Figure 4A) separates the progenitors (left-most, cyan), the erythrocytes (top, purple) and an internal branch of granulocyte/monocyte progenitors (bottom middle, brown). However, it fails to separate the megakaryocyte branch (top middle, blue) and groups dendritic cells (bottom right, red) together with basophils (green) and monocytes (orange). Furthermore, it groups neutrophils together with monocytes (right, magenta). MERLoT (Figure 4B) achieves almost pure megakaryocyte and dendritic cell branches and separates the bulk of the neutrophils from the monocytes (also see Supplementary Figure S21C). Additionally, it separates most of the neutrophils and basophils from the bulk of the progenitor population (see Supplementary Figure S21C). Although MERLoT better resolved certain branches in the lineage tree, neither method succeeds in separating basophils from monocytes and granulocyte-monocyte progenitors, and both trees contain a branch with a mixture of almost all cell types, including pluripotent progenitors (right, middle).

### Tree reconstruction performance assessment on synthetic data

In order to assess the quality of MERLoT’s lineage tree reconstruction, we compared its performance to four tools with a similar approach to trajectory inference, namely unsupervised methods that produce branch assignments and assign a pseudotime to each cell: SLICER (27), Monocle2 (26), TSCAN (28) and Slingshot (29). Since our focus was on correctly predicting cell labels and cell pseudotime, we needed data from complex topologies with known intrinsic developmental time.

**Figure 5.**
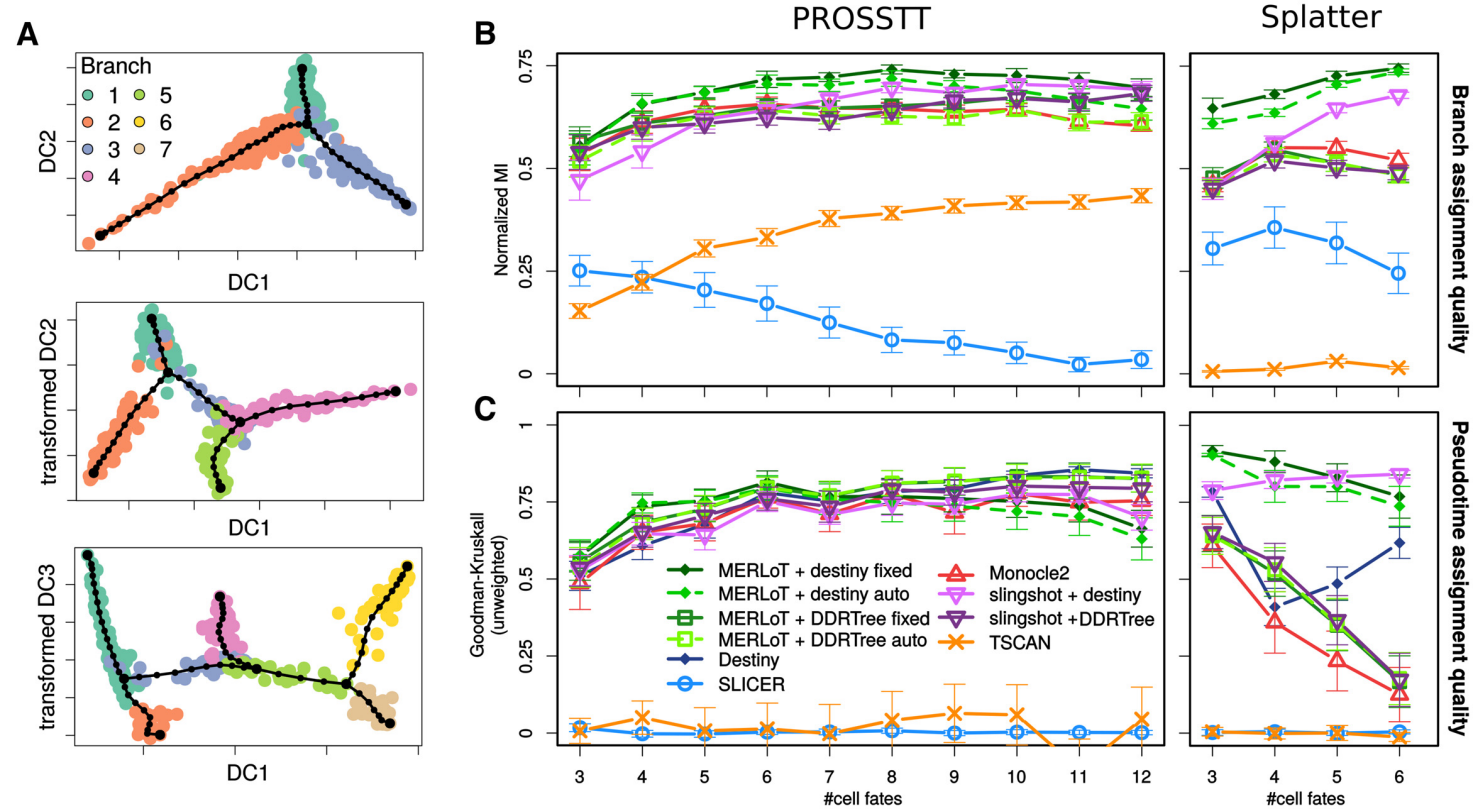
Simulated datasets and benchmarking: (**A**) Examples for diffusion map embeddings of PROSSTT simulations. From top to bottom: one, two and three bifurcations. Cell colors the labeled branch assignments. The double bifurcation is plotted by rotating diffusion components 2 and 3 around component 1. The triple bifurcation is plotted by rotating diffusion components 3 and 4 around component 1. (**B**) Branch assignment comparison using Monocle2, SLICER, TSCAN, Slingshot and MERLoT using both PROSSTT (left) and Splatter (right) simulations. Slingshot and MERLoT are used in combination with DDRTree and diffusion map (Destiny) coordinates. (**C**) Pseudotime assignment comparison. The error bars are 95% confidence intervals assuming the prediction scores are normally distributed.

For this purpose we developed PROSSTT (‘Materials and Methods’ section, (11)), a software that simulates scRNA-seq expression matrices with complex lineage tree structures. PROSSTT provides pseudotime and branch assignment labels for the cells, as well as branch connectivity information. Examples of diffusion maps for PROSSTT simulations and their lineage tree reconstructions with up to three bifurcations, performed by MERLoT, are shown in Figure 5A. Additionally, we used Splatter (‘Materials and Methods’ section, (12)), a suite to simulate cell populations with differential expression that can also be used to simulate branched topologies.

We generated three simulated datasets, two of 1000 simulations each by PROSSTT (‘lean’ and ‘deep’) and one of 1000 simulations with Splatter. All sets contain 10 subsets of 100 trees with 3–12 different cell fates and 1–10 bifurcations each. However, in the dimensionality reductions of datasets simulated by Splatter, branchpoints and endpoints often do not lie far enough from preceding tree segments. This makes it difficult for trajectory inference methods to correctly detect the tree structure, as they may connect non-adjacent tree segments, thereby creating ‘short-circuits’ (Supplementary Material, ‘Divergence Analysis’ and Supplementary Figure S18). Because of this reason, we only use Splatter simulations with 1–4 bifurcations for performance evaluation. The results for the higher order bifurcations and more analysis on why they were left out are shown in Supplementary material (Supplementary Figures S15, S4, S16 and S17; also see Supplementary Figure S9.)

For all simulations, we predicted the lineage trees, assigned cells to branches, and calculated cell pseudotime values using the aforementioned tools. Since MERLoT and Slingshot work on a given set of manifold dimensions provided by the user, we used them in combination with diffusion maps (provided by the Destiny package) or DDRTree coordinates (provided by Monocle2). For simplicity we will refer to these combinations as MERLoT_Destiny, MERLoT_DDRTree, Slingshot_Destiny and Slingshot_DDRTree. Additionally, MERLoT can be used with and without providing the correct number of cell fates (‘fixed’ and ‘auto’ modes). TSCAN and Slingshot do not provide a formal tree structure object, so in order to evaluate their performance we had to implement wrappers for them (see ‘Materials and Methods’ section).

#### Branch assignment quality

We assessed the agreement between predicted and labeled branch assignment predictions in the simulations using the Normalized Mutual Information (NMI), since it punishes splitting and merging clusters equally (Figure 5B). This avoids systematic advantages for methods that are biased to produce either more or less branches than the simulated ones. We also included other scoring measures (‘Materials and Methods’ section and Supplementary Figure S4).

In the left side of Figure 5B (top panel) we show the results for the ‘lean’ benchmark set. MERLoT_Destiny consistently outperforms Monocle2 and has the best overall performance, while Slingshot_Destiny achieves comparable results for more complex topologies. SLICER scores low mainly because its recursive branch assignment function crashes for many datasets or does not finish in less than 60 min, especially for complex topologies. TSCAN applies dimensionality reduction based on PCA which mixes up all cell types when projected in the reduced space and hence cannot be correctly classified.

In the Splatter benchmark set (Figure 5B, right) we only use lineage trees with up to four bifurcations to avoid short-circuits (Supplementary Figure S17). Still, tools with a DDRTree embedding perform worse than in the ‘lean’ set. Slingshot_Destiny again improves for more complex topologies but MERLoT_Destiny performs better. SLICER and TSCAN still underperform for the same reasons.

With the 10-fold increase in cell numbers in the ‘deep’ set we expected an overall increase in the performance levels of the methods. Indeed, this is the case for MERLoT, which is consistently between 5 and 10 percentage points better than in the original benchmark. Monocle2 performs at the same level as on the ‘lean’ set. Slingshot_Destiny shows improvement until five bifurcations, where performance starts deteriorating. This happens because starting at five bifurcations (5500 cells) Slingshot fails to compute distances between clusters for multiple simulated datasets due to a ‘computational [matrix] singularity’ error. This bug can be circumvented by adding small amounts of random noise to the diffusion map coordinates, in which case Slingshot’s performance increases with the complexity of the topologies (dotted line).

#### Pseudotime assignment quality

**Figure 6.**
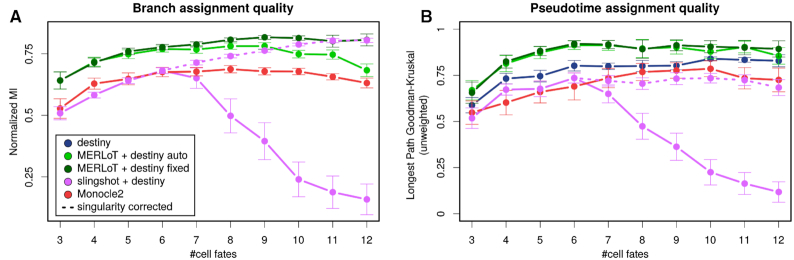
‘Deep’ benchmark set: Branch assignment (left) and pseudotime prediction (right) performance in the ‘deep’ PROSSTT benchmark. Slingshot and MERLoT are used only in combination with diffusion map (Destiny) coordinates, since this combination performed best in the ‘lean’ benchmark. The error bars are 95% confidence intervals assuming the prediction scores are normally distributed.

In a multi-branched lineage tree, multiple trajectories exist between progenitors and differentiated cell fates. Pseudotime only assigns an ordering within each trajectory, while pseudotime values are not comparable between non-consecutive tree branches. We therefore test pseudotime orderings on the cells that belong to the longest possible trajectory in terms of pseudotime steps in every simulation. We use the Goodman–Kruskal’s gamma (Figures 5C and 6B) and other indices (see ‘Materials and Methods’ section and Supplementary Figure S6) as a measure of concordance between the true and predicted orderings along the longest trajectory.

In the ‘lean’ set (Figure 5C, left), MERLoT_Destiny and Slingshot_Destiny perform better for easier topologies and are overtaken by MERLoT_DDRTree and Slingshot_DDRTree for more complex ones. MERLoT_DDRTree is overall the best method, and only gets slightly overtaken by Destiny for the most complex topologies in the benchmark. The poor performance of TSCAN and SLICER is a direct result of their poor performance at branch assignment.

In the Splatter set performance drops with the growing number of short-circuits, something that impacts Monocle2 considerably. Approaches based on diffusion maps, on the other hand, thrive.

The situation is clearer in the ‘deep’ set. MERLoT improves dramatically compared to the ‘lean’ set, reaching an improvement of 25 percentage points for topologies with 10 bifurcations. Destiny and Monocle2 perform at the level of the ‘lean’ set. Slingshot_Destiny does not improve in pseudotime compared to the original benchmark, even when the computational singularities are circumvented (‘singularity corrected’ in Figure 6B).

### Post lineage inference analysis

One of MERLoT’s assets is that it can reconstruct imputed gene expression profiles to study how a gene varies along the different paths in the lineage topology. These profiles interpolate and denoise the gene expression values of cells that are assigned to equivalent pseudotimes and facilitate the study/analysis of gene expression regulation, for example via detecting modules of genes that have correlated expression profiles. By using the imputed gene expression values recovered from MERLoT downstream analysis like Gene Correlation Network (GRN) reconstruction could be improved. Building GRNs requires identifying causality for the gene–gene interactions, which exceeds the scope of this work. However, as proof of concept, we derived a Gene Correlation Network (GCN), a proxy for a GRN. We analysed the dataset in which Treutlein and coworkers studied the transdifferentiation process of fibroblasts into neurons (20). Overexpression of the proneural pioneer factor *Ascl1* causes cells to exit the cell cycle and re-focus gene expression through distinct neural transcription factors. However, later on in the process a myogenic program competes with the neural one, producing undesired myocyte-like cells and lowering the efficiency of the direct reprogramming process.

**Figure 7.**
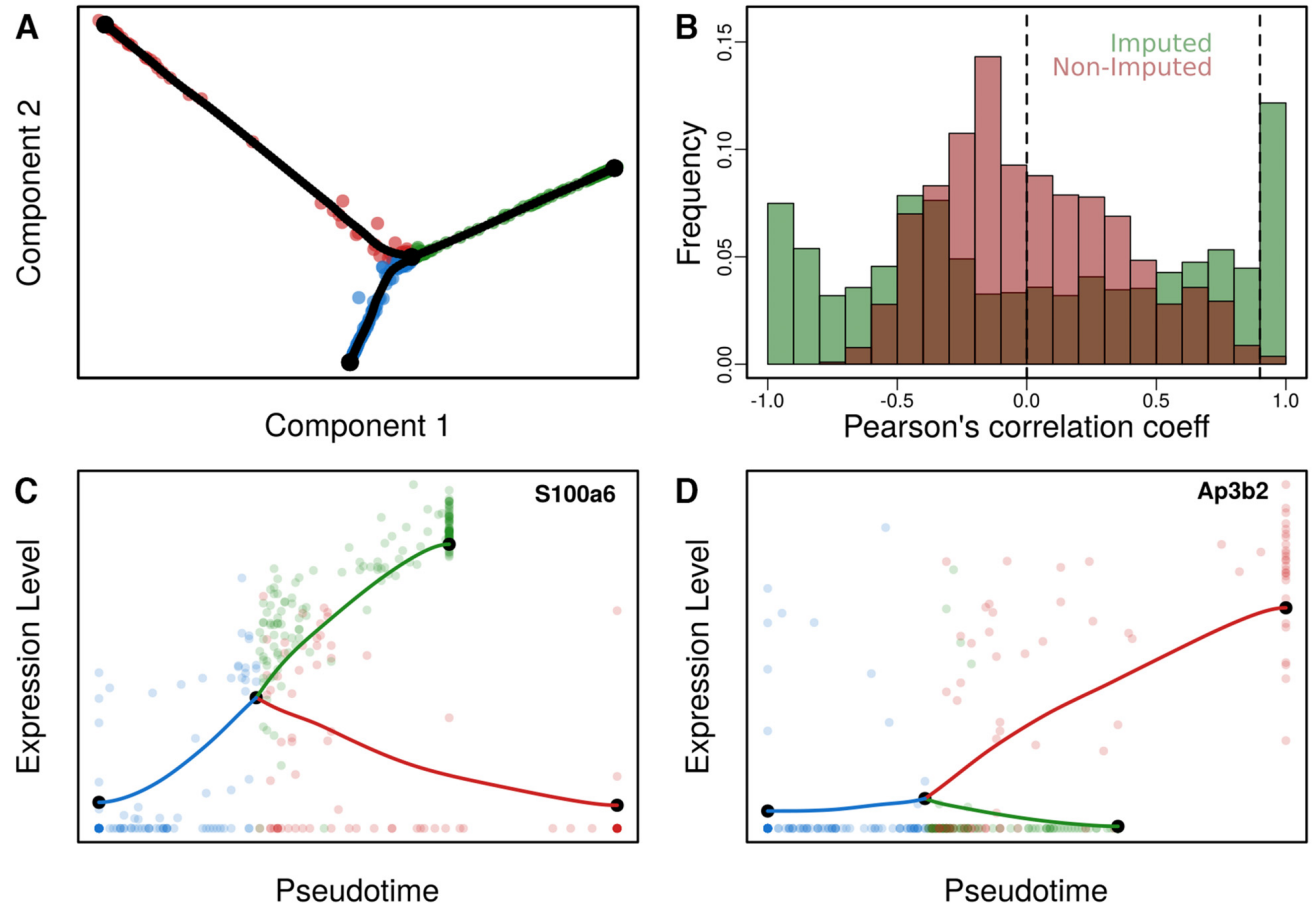
Lineage tree reconstruction of fibroblasts to neurons transdifferentiation: (**A**) Two-dimensional diffusion map embedding of cells together with the reconstructed lineage tree (tree support nodes shown in black). We observe a single bifurcated tree containing three branches corresponding to fibroblasts, neurons and myocytes. (**B**) Pairwise Pearson’s correlation coefficients for gene expression profiles using imputed (green) and non-imputed (red) values. The dashed line at *x* = 0 represents the separation between positively and negatively correlated genes. The dashed line at *x* = 0.9 points at the threshold for reconstructing the GCN in Figure 8. (**C**) Pseudotime gene expression profile of differentially expressed gene in myocytes. (**D**) Pseudotime gene expression profile of a differentially expressed gene in neurons.

We reconstructed the lineage tree from the data, which shows a single bifurcation (Figure 7A). Then, we embedded the tree structure into the gene expression space and recovered the imputed gene expression values for the support nodes. We calculated the Pearson’s correlation coefficients between all pairs of genes using both the original gene expression values from the cells and the imputed ones from the tree support nodes. In Figure 7B we show the Pearson’s correlation coefficient distributions for imputed and non-imputed gene expression values. While the non-imputed values concentrate most values between −0.5 and 0.5, the distribution of imputed values contains two subpopulations of genes close to −1 (highly anti-correlated) and 1 (highly correlated) separating them from the rest of weakly correlated genes. In Figure 7C and D we show the gene expression values of S100a6, a gene differentially expressed in myocytes and of Ap3b2, a gene differentially expressed in neurons, respectively.

**Figure 8.**
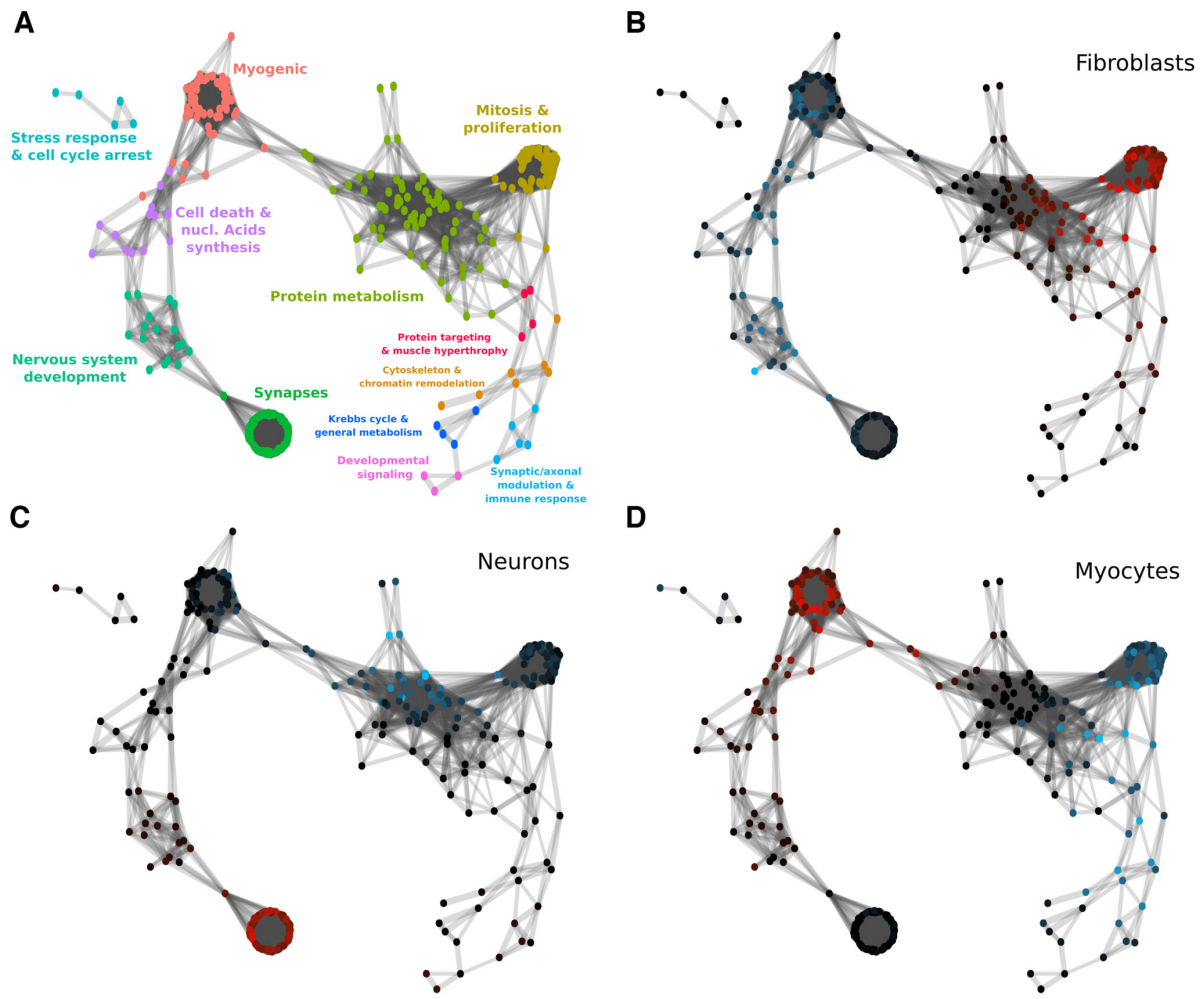
Reconstructed gene association network: (**A**) Gene clusters given the network connectivity were calculated and colored. Enriched GO terms were retrieved for each cluster and a general label summarizing their main implications are assigned to each cluster. (**B**) Differentially expressed genes in the fibroblast branch. (**C**) Differentially expressed genes in the neurons branch. (**D**) Differentially expressed genes in the myocytes branch. Genes that are differentially expressed with an *e*-value *e* < 10^−3^ are colored according to the mean difference in expression, shades of blue indicating downregulation and shades of red upregulation. Intensity corresponds to log fold change of gene expression (see color scale).

Once all pairwise gene expression correlations have been calculated, we built a graph where nodes correspond to genes and the pairwise Pearson’s correlation coefficients of imputed expression levels represent the weight of the edges connecting them. In Figure 8 we observe the graph that results from applying a layout (see ‘Materials and Methods’ section) to distribute highly correlated genes (Pearson’s correlation coefficients >0.9) in space. On this representation, close proximity corresponds to high correlation and *vice versa*.

For the quality of the reconstructed GCN, concentrating on highly correlated genes and using imputed gene expression levels is crucial. A low correlation threshold will add noise to the GCN by including false positive interactions. Additionally, the noise in the non-imputed data obscures the similarities between the time-dependent expression of genes, resulting in low correlation values (Figure 7B). This means that using a high-correlation threshold will result in a GCN with few, small connected components, while a lower threshold will inevitably lead to densely connected ‘hairy ball’ constructs that are difficult to interpret when raw, non-imputed values are used instead (Supplementary Figures S10–12).

After obtaining the GCN, we clustered the network (see Methods) and recovered the gene ontology (GO) terms that were enriched in each cluster (see ‘Materials and Methods’ section). In Figure 8A we show the reconstructed network colored by cluster and labeled according to the keywords that best represent their enriched GO terms. We used MERLoT’s module for finding differentially expressed genes, both upregulated (red) and downregulated (blue), on each of the three subpopulations assigned to each branch that composes the lineage tree structure (see ‘Materials and Methods’ section). This was done for each branch, i.e fibroblasts (Figure 8B), neurons (Figure 8C) and myocytes (Figure 8D). Genes that are differentially upregulated in the fibroblasts branch mainly belong to the clusters enriched for GO terms related with ‘mitosis and proliferation’ and ‘protein metabolism’. Downregulated genes in fibroblast cells mostly belong to clusters associated with the GO terms ‘myogenic’, ‘cell death and nucleic acids synthesis’ and ‘nervous system development’. We observe that for neurons, genes related to synaptic GO terms are upregulated while for myocytes the same happens for genes belonging to the myogenic cluster. Interestingly, downregulated genes on each branch belong to interconnected clusters. While neurons downregulate genes mostly associated with protein metabolism, myocytes downregulate genes associated with mitosis and proliferation, protein targeting and muscle hypertrophy and cytoskeleton and chromatin remodeling.

This dataset contains two mutually exclusive cell lineages, so gene expression patterns are mostly mirrored (upregulated genes in neuronal branch are downregulated in myogenic and *vice versa*). However when the tree topology is more complex, multiple transcriptional programs run in parallel and genes can have quite different behaviors in the different branches of the lineage tree. If GCNs are built using all cells together, spurious correlations can be recovered because of the so called Simpson’s paradox (3). An analysis of this effect is shown in Supplementary Note 5, where we reconstruct GCNs for the Guo dataset (Figure 2B)) that contains one progenitors population (zygote cells) and three mature cell types (TE, EPI and PE). We show that GCNs that are built using all cells do not allow a clear separation of gene markers that are specific for the EPI and PE lineages that emerge from the Inner Cell Mass (ICM) lineage.

## CONCLUSION

As single-cell RNA sequencing is becoming a mainstream technology, many datasets with highly complex underlying lineage trees will need to be analyzed. Here we have presented MERLoT, a tool to reconstruct complex lineage tree topologies in a more accurate way than other methods. We show this by applying MERLoT to various published datasets, but also by extensively testing its performance on a total of 2400 simulated datasets, produced by PROSSTT and Splatter. In this benchmark, MERLoT compares favorably to the state of the art in branch detection and pseudotime prediction using a variety of established performance indices.

Lineage tree reconstructions are not a final objective but rather a proxy to study changes in gene expression and understand the delicate regulation procedures that lead to organisms development, cellular differentiation, transdifferentiation and tissue regeneration. MERLoT simplifies and enhances downstream analysis in multiple ways. By utilizing an explicit tree structure, selecting subgroups of cells that belong to different tree segments or finding differentially expressed genes becomes straightforward. Apart from deriving the tree, MERLoT also imputes and interpolates gene expression by exploiting the use of EPTs in the high dimensional gene expression space applying the tree structure learned in the low dimensional embedding, drastically reducing noise and alleviating the problem of gene dropout and hence enhancing downstream analysis. A recent publication described how diverse connective tissue cell types regenerated axolotl limbs after amputation by converging to the homogeneous transcriptional signatures of multipotent progenitor cells (30). The authors used MERLoT to reconstruct the lineage tree, impute the gene expression values as a function of pseudotime, and study how gene expression levels changed among the different groups of cells in the process. Here, we have derived a GCN for a dataset representing fibroblast to neuron transdifferentiation as well as zygote to blastocyst differentiation (see Supplemental Note 5). We show that using MERLoT’s imputed expression values improves capture of gene–gene correlations, and could be used as input for more sophisticated methods that aim to reconstruct gene regulatory networks.

While our benchmark showed that MERLoT’s default approach leads to satisfactory results for a wide variety of topologies and expression matrices of various sizes, we are aware that when dealing with real data most methods do not work out of the box. Currently, researchers rely on external, expert knowledge about the studied systems and manually optimize strategies on every step of the process (gene selection filtering, cell quality filtering, different manifold embeddings and tuning of parameters related to all of these). MERLoT is flexible enough to allow supervision at different parts of the analysis pipeline, while providing default strategies that are robust enough to be used for exploratory analysis.

Single-cell RNA sequencing enables the study of time-dependent processes in unprecedented detail. With the help of tools like MERLoT, we can overcome the high noise and non-linearities in the data, reconstruct the cellular lineage trees and follow the change of gene expression over developmental time. Such time course gene expression profiles pave the way for the reconstruction of gene regulatory networks and eventually their quantitative modeling, which will profoundly advance our understanding of developmental processes.

## DATA AVAILABILITY

The 10 simulation sets with 100 simulated differentiations each are available at http://wwwuser.gwdg.de/∼compbiol/merlot/. The code necessary to run the benchmark on the simulations as well as instructions about how to set up a similar benchmark are available at https://github.com/soedinglab/merlot-scripts. Formatted expression data for the three datasets in Figure 2 are available at: https://github.com/soedinglab/merlot/tree/master/inst/example/

## Supplementary Material

gkz706_Supplemental_File
