# Supplementary material for: Reconstructing complex lineage trees from scRNA-seq data using MERLoT
Source: Nucleic Acids Res. 2019 Aug 20;47(17):8961–74. doi: 10.1093/nar/gkz706 (PMC6755101; doi:10.1093/nar/gkz706)
Supplement: gkz706_Supplemental_File [file gkz706_supplemental_file.pdf]

Supplementary Information
Reconstructing complex lineage trees from scRNA-seq data using MERLoT

R. Gonzalo Parra[1,2,†,*], Nikolaos Papadopoulos[1,†], Laura Ahumada-Arranz[1], Jakob El Kholtei[1], Noah Mottelson[1], Yehor Horokhovsky[1], Barbara Treutlein[3], and Johannes Soeding[1,*]

[*]corresponding author
[1]†equal contribution
[1]Quantitative and Computational Biology Group, Max Planck Institute for Biophysical Chemistry, Am Fassberg 11, 37077, Goettingen, Germany
[2]Genome Biology Unit, European Molecular Biology Laboratory, Meyerhofstraße 1, 69117, Heidelberg, Germany
[3]Department of Evolutionary Genetics, Max Planck Institute for Evolutionary Anthropology, Deutscher Platz 6, 04103, Leipzig, Germany

Methods

Suppl. Note 1 Lineage Tree Reconstruction by MERLoT

Given an expression matrix with N cells as rows and G genes as columns, a manifold embedding can be performed using techniques like Diffusion Maps or DDRTree embedding. Subsequently, informative dimensions can be kept in order to reduce dimensionality. Once cells are embedded into the low dimensional space, MERLoT can perform a lineage tree topology reconstruction following three steps which will be detailed in the following sections: (1) Calculating a Scaffold Tree with the location of endpoints, branchpoints and their connectivity. (2) Smoothing of the Scaffold Tree by using an EPT in the low dimensional manifold. (3) Embedding the EPT into the high-dimensional gene expression space and assigning pseudotime values to the cells.

Tree topology, is the set of endpoints and branchpoints that constitute the tree structure of the tree, i.e, a linear trajectory is only composed of a single branch with two endpoints and no branchpoints. A single bifurcated topology contains 3 branches that are connected via a branchpoint.

branches: set of connected nodes (cells) that constitute a branch that is defined as a linear segment of the tree contained between two endpoints or branchpoints. endpoint: is the node on which a branch ends. branchpoint: it is a node that connects three or more branches (in case of binary bifurcations or higher order multifurcations).

Scaffold Tree Reconstruction

Matrix of shortest paths between cells: We used Dijkstra's shortest path algorithm as implemented in the `csgraph` module from the `scipy` python library to calculate the shortest paths between all pairs of cells i and j based on their squared Euclidean distance d_{ij}^2. We use d_{ij}^2 instead of d_{ij} given that in a fully connected graph the shortest path between two nodes, based on d_{ij}, corresponds to the direct path between them. The `csgraph shortest_path` function returns the length D_{ij} of the shortest path that connects cells i and j. We extended the function to also return the number of cells S_{ij} on the shortest path connecting cells i and j. The modified code for `csgraph` is available at `github.com/soedinglab/csgraph_mod`.

Dijkstra's shortest-path-first algorithm:
Given a graph G where the distance between two nodes u, v is given by $dist(u, v)$, the algorithm will find the shortest paths from an initial origin node to all other nodes n in the graph. The algorithm initializes all nodes (except the origin) as unvisited; their distance $D[n]$ from the origin is infinite, and the distance of the origin to itself is zero. The origin is the current node c.

Consider all of the neighbours v of c, and calculate their distances from *origin* through the current node. If $D[c] + d(c, v) < D[v]$, then update $D[v]$ to $D[c] + d(c, v)$ and add c as the parent node of v. Repeat this until all nodes are visited. Afterwards, the shortest path that leads from node n to the origin is found by following the parent nodes of n until the origin is reached.

Endpoints search: MERLoT does not require users to define a starting point in order to reconstruct the tree topology. The first two endpoints in the tree correspond to the pair of cells k, l that maximize the number of cells S_{kl} on the shortest path between them (Fig. S1A): $(k, l) = \arg\max\{S_{kl} : 1 \leq k \leq l \leq N\}$. In case of ties, the pair of cells with the longest shortest path distance will be selected.

The next endpoint to be selected will correspond to the cell n that maximizes $s_{\mathcal{E}}(n)$, the number of cells being added to the scaffold tree structure. To compute $s_{\mathcal{E}}(n)$ we note that the new endpoint n must branch off from an internal node (cell) of the tree whose next-neighbour nodes must lie on the path of two already selected endpoints k and l. Therefore, the increase in number of cells is 0.5 times the minimum of the cells between k and l *via* n minus the cells between k and l, minimized over all pairs of endpoints (k, l) in the set of already selected endpoints $\mathcal{E}$.

$$s_{\mathcal{E}}(n) := 0.5 \times \min\{S_{kn} + S_{nl} - S_{kl} : k, l \in \mathcal{E}\}. \tag{1}$$

Stop criterion for endpoint search: In auto mode every time a new endpoint is selected we evaluate if $\max\{s_{\mathcal{E}}(n',) : 1 \leq n' \leq N, n' \notin \mathcal{E}\} > \sqrt{N}$ holds true. Otherwise, we calculate the branchpoints and tree connectivity for the endpoints in $\mathcal{E}$, including n, using the methodology explained in the next subsection. After this, all cells are mapped to their closest branch. If the branch added by the selected n endpoint contains more than `MinBranchCells`= $\sqrt{N}$ cells mapped to it, the branch is kept in the tree scaffold structure and the endpoints search is repeated. Otherwise, the endpoint search terminates and n is discarded as endpoint. The `MinBranchCells` threshold can be modified by the user. Alternatively, instead of using a stop criterion, users can set the number of endpoints that are aimed to be found (fixed mode) regardless of the branch lengths.

Branchpoints search and tree connectivity definition

Once endpoints are found, we apply the in combination with a heuristic criterion to find the cells that best represent the branchpoints in the tree structure. By doing this, we also detect the tree connectivity among endpoints and branchpoints

Given a set of endpoint and branchpoint cells we iterate the following steps: (1) Use the Neighbour Joining (NJ) criterion [1] to select the pair of (k, l) cells that will be joined *via* a branchpoint next. 2) Find the cell m that best represents the branchpoint between k and l and add the $l - m$ and $m - k$ edges to the tree (Fig. S2).

Let $\mathcal{V}$ be the set of yet unprocessed endpoint and branchpoint nodes of the tree. We initialize $\mathcal{V} \leftarrow \mathcal{E}$ with the endpoint set $\mathcal{E}$ determined in the previous subsection.

(1) Given the matrix D_{kl} of shortest path lengths between nodes in $\mathcal{V}$ (section 1.1), the NJ criterion allows us to pick two nodes k, l in $\mathcal{V}$ that are guaranteed to be next neighbours and therefore can be linked via a single branchpoint. The nodes to be joined are chosen such that $(k, l) = \arg\min d^{\mathrm{NJ}}_{k,l}$ where d^{NJ}_{kl} is the neighbour-joining distance,

$$d^{\mathrm{NJ}}_{kl} := D_{kl} - \frac{1}{|\mathcal{V} - 2|} \sum_{m \in \mathcal{V}} (D_{mk} + D_{ml}) \,. \tag{2}$$

(2) We determine the branchpoint cell m as the one that minimizes the sum of distances to nodes k, l and the mean distances to all other nodes included in $\mathcal{V}$,

$$m = \arg_m \min \left\{ D_{km} + D_{lm} + \frac{1}{|\mathcal{V} - 2|} \sum_{n \in \mathcal{V} \setminus \{k,l\}} D_{nm} \right\} . \tag{3}$$

Next, k and l are removed from $\mathcal{V}$ and the new branchpoint m is added to $\mathcal{V}$, $\mathcal{V} \leftarrow \mathcal{V} \setminus \{k, l\} \cup \{m\}$.. Also, the edges $l - m$ and $m - k$ are added to the tree (Fig. S2).

We iterate (1) and (2) until $|\mathcal{V}| = 2$, which means no further branchpoints exist. After termination, we have determined the tree topology with its $|\mathcal{E}|$ endpoints and $|\mathcal{E}| - 2$ branchpoints, each represented by a cell. The same cell can be detected more than once as a branchpoint and hence bifurcations with higher orders than binary ones are possible.

Suppl. Note 2 Scaffold Tree pseudocode

```
INPUT
CellCoordinates // N: number of cells, G: number of components

// 1. Calculate cell pairwise squared euclidean distances.
$D_e^2$ = CalcEuclideanDistances(CellCoordinates)^2

// 2. For every pair of cells c_i, c_j calculate:
```

```
// Shortest path euclidean distance D_p(c_i, c_j)
// Shortest geodesic distance, D_g(c_i, c_j)
(D_p, D_g) = DijkstraShortestPath(D_e)

// 3. Calculate endpoints E
// a) First pair of endpoints: Find pair of cells k, l with maximum
// geodesic distance D_g
(k,l) = argmax(D_g)
E = list()
E.append(k), E.append(l)

// Find further endpoints
// Stop criteria depends on the MERLoT flavor - either stop after a certain number
// of branches has been reached (fixed mode) or after new branches add less cells
// than a cutoff.
while stop_criteria == False:
  // initialize vector containing endpoint score
  s_E = zeros(N)
  for n in N:
    // calculate how many cells would be added to the tree if
    // n was a new endpoint, in terms of shortest paths
    aux = zeros(length(E), length(E))
    for e_1 in E:
      for e_2 in E:
        aux[e_1, e_2] = D_g[e_1, n] + D_g[n, e_2] - D_g[e_1, e_2]

    s_E[n] = 0.5 * min(aux)
    E.append(max(s_E))

// 4. Find branchpoints and define topology edges in list B
// Initialize V which will contain the remaining endpoints to be joined during each step
V = E
B = list()
// List of edges. We need this for the connectivity.
Edges=list()
while length(V) >= 2:
  // find pair of endpoints to be joined
  for e_1 in 1:length(V):
    for e_2 in 1:length(V):
      // calculate Neighbour Joining distance between endpoints e_1 and e_2 according
      // to equation (2) in Materials and Methods
      $D_NJ[e_1, e_2] = calc_NJ_distance(V[e_1], V[e_2])

  // endpoints to be joined
  (e_1, e_2) = min(D_NJ)
  // select cell m that minimizes D_NJ as branchpoint between endpoints
  // e_1 and e_2 according to equation (3) in materials and methods
  b = argmin(D_NJ[e_1, e_2])

  B.append(b)

  // Add edges to tree topology
  Edges.append([e_1, b])
  Edges.append([e_2, b])

  // replace joined endpoints from V with b
  V.remove(e_1)
  V.remove(e_2)
  V.append(b)
```

```
// no further branchpoints to be searched, append the last edge to the tree topology.
Edges.append (V[1], V[2])
return(E, B, Edges)
```

Elastic Principal Tree in the Low Dimensional Manifold

We use the coordinates of the endpoints and branchpoints of the scaffold tree and their connectivity to initialize the EPT. Further nodes are then added by the EPT algorithm by iterative bisection of edges until the specified number of k support nodes is reached. This procedure cannot modify the number of endpoints and branchpoints specified at the initialization step.

In every iteration an energy potential defined as follows is minimized:

$$U = \text{MSE} + \mu U_E + \lambda U_R. \tag{4}$$

MSE (mean squared error) is the sum of squared distances of cells to their closest tree support nodes. U_E and U_R are two regularization terms that ensure that we learn trees with regularly spaced points and with edges without kinks, respectively.

We performed a grid search around the EPT default values in order to optimize μ and λ and visually examined the reconstructed EPTs on the datasets shown in Fig. 2. Using $k = 100$, we obtained $\mu_0 = 0.0025$ and $\lambda_0 = 0.8 \cdot 10^{-9}$. If the number of nodes used for calculating the EPT is changed, μ and λ are adjusted according to $\mu = (k-1)\mu_0$ and $\lambda = (k-2)^3\lambda_0$. All reconstructions in our benchmark were performed with the standard function using $k = 100$. However, for some particular topologies μ and λ might need to be tuned in order to produce optimal results, in particular if k is increased a lot. Alternatively, MERLoT can bisect the edges in a given EPT, by additional nodes producing a new EPT with almost $2k$ support nodes. Note that these are special cases.

Scaling of μ and λ with the number of tree points K. We would like to be able to change K without changing the shape of the tree. Say we change from K tree points to $2K-1$. Then we want the even-numbered points $y'_0, y'_2, y'_4, \ldots, y'_{2K-1}$ to come to lie exactly where previously we had points $y_0, y_1, \ldots, y_{K-1}$. Since the data term will stay very nearly the same if the points were already close to each other, the bending and stretching energies should also stay the same. How do we need to scale $\mu(K)$ and $\lambda(K)$ in order to keep these terms the same?

Let us start with the stretching energy. The distances between neighbouring points will be half the previous distances and their squares will be a quarter. On the other hand, we will have 2K-2 such terms instead of $K-1$, so twice more. Therefore, if we scale

$$\mu(K) = (K-1)\mu_0\,, \tag{5}$$

the total stretching energy will be conserved.

Regarding the bending energy, the terms $||y_k - (y_{\text{pre}(k)} + y_{\text{suc}(k)})/2||$ can be approximated as $r_k(1-\cos(\theta_k/2))$, where r_k is the bending radius at point y_k and θ is the angle of direction change between y_{k-1} and y_{k+1} (which is equal to the angle between $\overline{Cy_{k-1}}$ and $\overline{Cy_{k+1}}$, where C is the center of the circle of the bending radius through y_{k-1}, y_k, and y_{k+1}). Since θ is halved upon going from K to $2K-1$ tree points, the distance $r_k(1-\cos(\theta_k/2)) \approx r_k\theta_k^2/8$ will be 4 times smaller and the squared distance will get 16 times smaller. Since there will be $2K-3$ instead of $K-2$ such squared terms, we need to scale

$$\lambda \approx (K-2)^3\lambda_0 \tag{6}$$

in order to keep the bending energy approximately constant.

Elasticity hyperparameters: We selected appropriate default elasticity hyperparameters for the elastic and embedded tree by performing a grid search around the default EPT hyperparameter values. If N_y is the number of nodes of the elastic tree, we define $\mu_{\text{el}} = N_y\mu_0$ and $\lambda_{\text{el}} = ((N_y-2)^3)\lambda_0$, with $\mu_0 = 0.0025$ and $\lambda_0 = 0.8 \cdot 10^{-09}$. For the embedded tree, we use $\mu_{\text{emb}} = (N_y-1)\cdot\mu_0\cdot\phi_\mu$ and $\lambda_{\text{emb}} = (N_y-2)^3\cdot\lambda_0\cdot\phi_\lambda$, where $\mu_0 = 0.00625$, $\lambda_0 = 2.03 \cdot 10^{-09}$, and $\phi_\mu = \phi_\lambda = 20$. In the benchmark, where N_y was 100, this amounted to values of $\mu_{\text{el}} = 0.0025$, $\lambda_{\text{el}} \approx 0.00075$, $\mu_{\text{emb}} = 12.375$, and $\lambda_{\text{emb}} \approx 0.038$. The absence of wiggles in 2D or 3D projections of the tree and gene expression profile plots strongly indicate that the trees did not overfit the cell density.

In the "deep" benchmark, beyond the default values we also tested MERLoT with the default ElPiGraph.R hyperparameter values for the elastic tree functions, $\mu_{\text{ElPi}} = 0.1$ and $\lambda_{\text{ElPi}} = 0.01$. Performance in pseudotime prediction is indistinguishable for both approaches (Fig. S7, right panel). In branch assignment, while the elastic trees (red, cyan) perform at similar levels, the ElPiGraph.R embedded trees tend to have higher average scores for higher order topologies.

After the benchmark was finished we noticed that when using the ElPiGraph.R parameters MERLoT produced embedded trees with many wiggles, indicative of overfitting. We explore this in more detail in two R notebooks (`http://wwwuser.gwdg.de/~compbiol/merlot/examples/`), using data from [2] and [3], respectively.

Suppl. Note 3 Benchmark on Synthetic Datasets

Suppl. Note 3.1 Simulating count data of branching processes with PROSSTT

PROSSTT generates a simulated scRNA-seq dataset in four steps:

1. Generate tree: We sample the number of genes from a discrete uniform distribution between 100 and 1000. These are typical numbers left in real datasets after filtering out uninformative genes.

Each tree segment in every simulation had a pseudotime length of 50, corresponding to 50 cells on the branch (in homogeneous sampling model). This length allows the expression programs to diffuse enough to be distinct from each other. Starting at 2 bifurcations, alternative segment connectivity possibilities become available (Fig. S8)). We chose the lineage tree topology randomly for every simulation.

2. Simulate average gene expression along tree: PROSSTT models relative gene expression as a linear mixture of a small number of expression programs. For each tree segment, we simulate the time evolution of expression programs as a random walk with momentum term. Each simulation uses $K = 5b + u$ expression programs, where b is the number of branchpoints and u is drawn from a uniform integer distribution $\mathcal{U}\{3, 20\}$. Each program contributes to the expression of every gene, with weights drawn from a gamma distribution (shape parameter a scales inversely with number of programs, rate parameter b is 1). A scaling factor (library size) was sampled for each cell and multiplied to the average gene expression values. We used a log-normal distribution with $\mu = 0$ and $\sigma = 0.7$.

3. Sample cells from tree: PROSSTT can sample cells in the tree according to a given density function. Here we used a uniform density and drew $50 \times b$ cells, where b is the number of branches.

4. Simulate UMI counts: We simulate UMI counts using a negative binomial distribution. Following [4] and [5], we make the variance σ_g^2 depend on the expected expression μ_g as $\sigma_g^2 = \alpha_g \mu_g^2 + \beta_g \mu_g$. The α_g and β_g values were sampled from log-normal distributions with $\mu_{a_g} = 0.2, \sigma_{a_g}^2 = 1.5$ and $\mu_{b_g} = 2, \sigma_{b_g}^2 = 1.5$ respectively. These values are typical for single-cell RNA sequencing UMI counts. For more information about default parameters choices and the algorithm, please refer to [6].

We provide the scripts used to create the simulations (`https://github.com/soedinglab/merlot-scripts`) as well as the simulations themselves (http://wwwuser.gwdg.de/∼compbiol/merlot/).

Suppl. Note 3.2 Simulating count data of branching processes with Splatter

We chose Splatter simulation hyperparameters to mirror those in the PROSSTT simulations as closely as possible.

- **Global parameters:** the number of genes was picked from the same discrete uniform distribution $\mathcal{U}\{100, 1000\}$. A random seed was set and included to ensure reproducibility.
- **Batch parameters:** The number of batch cells (substitutes total number of cells if only one batch is present, as is the case here) was set to $100 \times b$, where b is the number of branches. This is twice the number sampled for PROSSTT simulations, which increased the robustness of the dimensionality reduction. The other batch parameters were left to their default values.
- **Group parameters:** The number of groups was set to the number of branches, and the occurrence probability of each group was set to $1/b$.
- **Differential expression parameters:** Since we were only interested in simulating informative genes, we set the probability of differential expression per gene to 1. All other parameters were left at their default values.
- **Differentiation path parameters:** The topology of the lineage tree was input as a vector of originating points per branch (`path.from` parameter). Branch lengths were set to 50 via `path.length`.

Suppl. Note 3.3 Assessing Methods Performance

Tree reconstruction tools need to succeed at two intertwined tasks: to arrange all cells according to their internal developmental time, while also detecting and separating different branches of the tree. Ideally, one would evaluate algorithm performance on both tasks at once. However, we were not able to find such a measure and so evaluate branch assignment and pseudotime prediction separately.

Branch assignment We treated branch assignment evaluation as a clustering problem. We can consider all cells mapped to a given branch of a tree as a cluster and compare the set of labels produced by PROSSTT with those that

are predicted by each algorithm. Given a cell c, let $Tr(c)$ be the cluster identity of c in the ground truth and $A(c)$ the cluster assigned to c by the algorithm. We define a pair of two cells c_i, c_j as a...

$$\begin{array}{lll} \text{true positive (TP)} & \text{if} & \text{Tr}(c_i) = \text{Tr}(c_j) \wedge \text{A}(c_i) = \text{A}(c_j) \\ \text{true negative (TN)} & \text{if} & \text{Tr}(c_i) \neq \text{Tr}(c_j) \wedge \text{A}(c_i) \neq \text{A}(c_j) \\ \text{false positive (FP)} & \text{if} & \text{Tr}(c_i) \neq \text{Tr}(c_j) \wedge \text{A}(c_i) = \text{A}(c_j) \\ \text{false negative (FN)} & \text{if} & \text{Tr}(c_i) = \text{Tr}(c_j) \wedge \text{A}(c_i) \neq \text{A}(c_j) \end{array}$$

Using these four values, many popular clustering indices can be computed (Fig S4): the F1 measure (Fig. S4A) is $2PR/(P + R)$, (where P is the precision $TP/(TP + FP)$ and R is the recall $TP/(TP + FN)$), the MCC (Fig. S4B) is

$$\frac{(TP \cdot TN - FP \cdot FN)}{\sqrt{((TP + FP)(TP + FN)(TN + FP)(TN + FN))}},$$

and the Jaccard Index (Fig. S4C) is $TP/(TP + FP + FN)$

While all the aforementioned indices and measures are well established, they are suboptimal performance indicators for the problem at hand, since they don't take cluster structure into consideration. As the simulated lineage trees become bigger and more complex, the number of cell pairs that are not in the same tree segment is going to grow much faster than the number of cell pairs in different tree segments. The number of TPs will grow much slower than the number of TNs, something that will, for example, inflate the the MCC index. Additionally, the number of possible FNs becomes much higher with each additional segment added to the tree, something that affects the Jaccard index and the F1 score. In short, these measures, while they produce consistent results, they don't take into account the number of clusters in the data, and as such are suboptimal descriptors of clustering performance. In our opinion the NMI is best for assessment of branch assignment, since it captures the amount of information present in the original clustering that was recovered by the prediction (values between 0 and 1). It corrects the effect of agreement between clusters that is due to chance by using a hypergeometric background distribution and punishes overbranching and merging branches almost equally [7]. Given the predicted and real cluster assignments U and V, NMI is defined as

$$\text{NMI}(U, V) = \frac{\text{MI}(U, V) - \mathbb{E}[\text{MI}(U, V)]}{\max\{H(U), H(V)\} - \mathbb{E}[\text{MI}(U, V)]}.$$

where $H(U)$ is the entropy of U, MI(U, V) is the mutual information of U, V and $\mathbb{E}$ denotes the expectation value under the null model of independent U and V.

Pseudotime prediction Evaluating the performance of the different methods consists of quantifying the degree of agreement between the true/labeled and the predicted orderings provided by the different algorithms. Pseudotime only establishes a partial ordering on cells and no absolute time. Therefore, cells on branches not passed through one after the other cannot be compared. We find the longest path in the tree (from the root to a leaf) and compare the predicted pseudotime with the simulated one for the cells on this path.

In this sense, pseudotime prediction assessment is a comparison of ordered sequences, and we follow the suggestions of [8] by using the Goodman-Kruskal index and the Kendall index (weighted and unweighted). All four indices count how many pairs of cells have been ordered correctly (S_+) or incorrectly (S_-) and produce similar results for the benchmark. The unweighted Goodman-Kruskal index is the simplest approach: $(S_+ - S_-)/(S_+ + S_-)$.

Complexity of predicted topologies We analyzed the predicted topology complexity, measured in number of branchpoints per lineage tree (Fig. S9). TSCAN and SLICER usually predict more branches than the ones being simulated. Monocle2, Slingshot, and MERLoT mostly overbranch for the simpler topologies but reverse the trend towards more complicated ones. The only notable difference between the "lean" and Splatter sets is that Slingshot_DDRTree detects more branches in "lean".

Suppl. Note 3.4 Benchmarked Methods and Parameters

Monocle2: Monocle2 (version 2.10.1) uses a reverse graph embedding (RGE) technique called DDRTree [9] to create a lineage tree and map points from the low-dimensional embedding of the tree to the original gene space. Based on Monocle's vignette, we used the `negbinomial()` expression family for the data and the proposed defaults for lower detection limit (1), minimum expression (`detectGenes` function, 0.1), mean expression threshold (gene ordering, 0.5), and empirical dispersion threshold ($2 \cdot dispersion\_fit$).

Generally, a successful dimensionality reduction will capture a topology with B bifurcations in its first $d = B + 1$ dimensions. While Monocle2 runs DDRTree by default on two dimensions, in our benchmarks we used $B + 1$ dimensions, as it performed better. DDRTree did not always return d dimensions; if fewer dimensions were provided we used the maximum possible number.

Monocle2 assigns a branch identity to each cell in the `State` column of the phenotypic data table (`pData`). It calculates pseudotime as distance from one of the endpoints of the lineage tree it produces. In order to pick the correct endpoint,

we checked if the cell with simulated pseudotime $t_0 = 0$ was in an outer branch; if true, the corresponding endpoint was assigned t_0. In the rare event that it was placed in an inner branch, we used Monocle's chosen starting `State` for pseudotime calculation.

SLICER: SLICER uses Locally Linear Embedding (LLE) to perform dimensionality reduction and uses a neighbour graph to order cells according to their distance from a user-specified starting cell (pseudotime prediction). It uses geodesic entropy to recursively detect branches.

We used SLICER version 0.2.0 (commit `cb1be8a`) by following the instructions that accompany the software on its github page (`https://github.com/jw156605/SLICER/`). We used the software's gene selection process as-is and used the selected genes to determine the best k value for the LLE, with a $k_{\min}$ value of 5. Much like with Monocle2, using $d = B+1$ LLE dimensions (over the default 2) for a dataset with B bifurcations improved performance. We used the same k value for the creation of the low-dimensional k-nearest neighbour graph as we did for LLE. For every simulation, we used the cell with labeled pseudotime $t_0 = 0$ as the starting point of the `cell_order` function, which predicts pseudotime for each cell. Finally, we used the same start point for the branch assignment step (`assign_branches`). This step very often failed to execute; these cases were assigned the worst possible score of each branch assignment measure. The issue was reported to the authors (`https://github.com/jw156605/SLICER/issues/7`) but until the time of this writing was not resolved.

Destiny: Destiny produces a dimensionality reduction and a pseudotime prediction based on it. We used the destiny diffusion map space as input for MERLoT, and benchmarked destiny's Diffusion Pseudotime (DPT). We used destiny (version 2.6.1) as described in the Bioconductor vignette. First we normalized the input data by correcting for library size and then log-transformed them. The only free parameter is the number k of nearest neighbours. By default, destiny uses a heuristic to determine it. For a dataset with B bifurcations we gave destiny $d = B + 1$ dimensions of the diffusion map to determine pseudotime, following the same reasoning as with Monocle2 and SLICER. We retrieved pseudotime predictions from destiny by calling the `DPT` (Diffusion Pseudo-Time) function on the diffusion map object and then using the dimension which corresponded to diffusion pseudotime distances from the cell with pseudotime $t_0 = 0$.

MERLoT: We ran different flavors of MERLoT on embeddings from different dimensionality reduction tools:

1. **MERLoT + diffusion maps:** destiny was run to produce diffusion maps for MERLoT. The same protocol as in section Suppl. Note 3.4 was used, except for the selection of free parameter k, which was done by a simple optimization. For a simulated lineage tree with B bifurcations we tested values of k between 5 and 100 and kept the k value that maximized the drop-off after the $d + 1$'th eigenvalue of the diffusion map, where $d = B + 1$.

2. **MERLoT + DDRTree:** Monocle2 was run in order to produce DDRTree coordinates. The number of coordinates to be used to reconstruct the lineage tree was selected as described in section Suppl. Note 3.4.

Each of these two options was combined with two different ways in which MERLoT finds the number of endpoints in the dataset.

1. **MERLoT auto:** MERLoT is run without the specification of the number of endpoints in the tree. MERLoT will use its internal branch length heuristic to determine new branches. The algorithm stops finding new endpoints when the new branch aimed to be included in the tree structure does not contain more than $\sqrt(N)$ number of cells, with N being the total number of cells in the dataset.

2. **MERLoT fixed:** MERLoT is run with a specified number of endpoints (tree leaves) to be found. MERLoT will ignore its internal branch length heuristic and will keep searching for branches until it reaches the specified number of endpoints regardless of how many cells are mapped to them.

All 2×2 combinations were tested (MERLoT on diffusion maps auto/fixed and MERLoT on DDRTree coordinates auto/fixed). The benchmark was performed with commit `9a9fc93` of the "scaffold" branch.

TSCAN: TSCAN (version 1.16.0) is a tool that groups similar cells into clusters and then creates an undirected minimum spanning tree (MST) that connects them, a different approach from the other tools assessed in this benchmark. We followed the typical TSCAN pipeline as presented in the tool's vignette on the Bioconductor site. This consists of a preprocessing step (`preprocess` function) followed by clustering and construction of the Minimal Spanning Tree (MST), which happen in the same step (`exprmclust` function). The `exprmclust` function accepts a number of clusters as parameter. If the user inputs a range of possible clusters, TSCAN will pick the number of clusters using the Bayesian Information Criterion.

TSCAN's cluster approach is an issue when comparing TSCAN predictions to simulations that are made using paths (such as PROSSTT or Splatter), since there is no 1-to-1 relationship between branches and the TSCAN clusters. Additionally, when run with default parameters, TSCAN very often predicts ambiguous topologies, where, because the MST is undirected, it is not possible to tell if bifurcations are present or not.

In order to address this concern we ran TSCAN with the maximum number of clusters possible. This was achieved by starting from 50 (a number well above the maximum number of branches in the simulations), and decreasing the number of clusters until a clustering is produced. Afterwards, co-linear clusters are collapsed and the cells of branching clusters are re-assigned to the nearest adjacent clusters.

Unfortunately, this creates other complications, since TSCAN only calculates pseudotime for the longest trajectory (path from endpoint to endpoint) in the tree. After consulting the authors (see `https://github.com/zji90/TSCAN/issues/4`), we ran the clustering step of TSCAN with 2 clusters. This guarantees a linear path that includes all the cells and thus a global pseudotime ordering can be obtained.

Slingshot: Slingshot [10] is a trajectory inference algorithm very similar to TSCAN. In a first stage, it creates a cluster-based MST to identify the key elements of the global structure - the branches and branching points. In a second step, it uses simultaneous principal curves (an extension of the principal curve algorithm proposed by [11]) to construct smooth trajectories that represent paths from the root of the lineage tree root to its leaves. It then assigns pseudotime to cells by projecting them on these principal curves.

Slingshot does not compute a reduced manifold nor does it perform the clustering step. It is left to the user to supply both. For this benchmark, we used the manifold reductions produced by the other tools (diffusion maps by destiny, DDRTree embedding from Monocle 2, PCA from TSCAN). For the clustering, we used `mclust` [12], an R package for model-based clustering. We ran the `mclustBIC` function, allowing the number of clusters to be between 5 and 50 (for the same reasons as TSCAN, see above). This function fits a Gaussian finite mixture model via an expectation-maximization (EM) procedure, and returns an optimal number of cluster means and variances. In the next step, the cells are assigned to the closest cluster centroid according to the EM model that maximizes the Bayes Information Criterion.

Slingshot assigns a pseudotime to each cell in every trajectory it detects. This means that when two trajectories are close to each other, some cells may have multiple pseudotime values. All trajectories start at the same cell though, so in cases where cells have multiple pseudotime values, these are usually very similar and can be averaged without problems, thus creating global pseudotime values for all cells.

Since Slingshot follows the same cluster strategy as TSCAN, it also faces the same problems when it comes to evaluation in the context of PROSSTT. To avoid biasing the benchmark against Slingshot, we followed the same strategy as above, by identifying and collapsing all co-linear clusters and reassigning cells to the branches that are created.

We used Slingshot version 1.0.0, available for Bioconductor 3.8.

During Slingshot runs on the "deep" benchmark we encountered problems with most of the high-order bifurcations. Specifically, Slingshot struggles when determining the cluster-to-cluster distance. The distance measure is $D^T SD$, where D is the difference of the cluster centroids and S is the matrix inverse of the sum of the covariance of each cluster. What happens frequently is that this inversion is impossible due to "computational singularity". For some cases, rerunning the analysis did not produce the same issue. For the rest, we added a small amount of random noise to the diffusion maps. This noise was sampled from $\mathcal{N}(0, d/100)$, where d is the difference between the minimum and maximum absolute value in manifold space. We reported the issue to the developers `https://github.com/kstreet13/slingshot/issues/35`.

Suppl. Note 3.5 Divergence analysis

While benchmarking method performance on data simulated with Splatter, we noticed that multiple methods did not perform according to expectations. The issue was particularly obvious in the evaluation of pseudotime prediction (Fig. S15D), where Monocle2 sank to the level of random predictions, and MERLoT, which, even though it excelled at branch assignment (Fig. S15B), dropped off to quite low accuracy for large numbers of bifurcations. Additionally, TSCAN, which in the PROSSTT benchmark proved to be competent in branch assignment (Fig. S15A), returned completely nonsensical predictions for data simulated by Splatter.

As these methods have all been applied successfully on real data, we chose to examine the simulations. By visual inspection of the manifold embeddings we observed that the manifold embeddings of Splatter simulations often presented "short-circuits", where parts of the lineage tree seemed to fold back to preceding tree segments, effectively creating cycles. While branches were often separated correctly (i.e. the cell clustering was correct), they were connected in wrong ways, decreasing the pseudotime prediction accuracy.

Since the manifold embeddings are a projection that aims to retain the most important dynamics in the data, we hypothesized that the reason for these short-circuits was that the offending branches did not diverge enough, or even converged towards previous tree segments. In terms of gene expression, this means that differentiating phenotypes (captured transcriptomes) were either not different enough or that they converged towards preceding phenotypes.

To test this hypothesis, we measured the distance of each waypoint (endpoint or branchpoint) from the origin, and normalized it by the average branch length in the path that led to it. The calculations described below were performed on the simulated gene expression data, after normalization for library size and log-transformation (see scripts `divergence_euclidean.R` and `divergence_diffmaps.R`).

As explained in Fig. S16, we retrieved the cells with minimum and maximum pseudotime in each branch (start cells and end cells respectively), calculated their pairwise distances, and defined their average d_b as the length of branch b.

Next, we took the cells with globally minimum pseudotime and calculated their distances from all end cells. This is the minimum direct distance d'_b of each branch b from the origin. We normalized each d'_b with $\bar{d}_b = \frac{1}{|p|}\sum_{b' \in p} d_{b'}$, where p is the path $[b_0, b_1, ...b]$ from the origin branch b_0 to branch b. Effectively, this yields the distance from the origin measured in average branch lengths. Pooling the normalized distances from all paths of equal length (especially since all branches have the same length in the PROSSTT and Splatter simulations) shows how much the change in expression values correlates with pseudotime.

The divergence curve of PROSSTT (Fig. S17A) shows what we expected: monotonic growth (i.e. longer paths are on average further away from the origin) and values above 1, indicating that paths with two branches or more consistently end outside a one-branch-length radius from the origin, something that reduces the possibility of wrong assignments from the methods.

On the contrary, the divergence curve of Splatter (Fig. S17B) stays almost completely below 1 and even shows a slightly negative slope. This means that expression profiles of cells in later differentiation stages don't move further from the origin with increasing pseudotime length. We believe this happens because Splatter does not include co-regulation in its differentiation simulation model. This leads to the differences between branches being completely random, and while this may work to separate two diverging branches from each other, it does not seem to yield realistic diverging cell lineage trees.

As a control, we performed the divergence analysis in the diffusion maps created by destiny for the benchmark (panels G,H in Fig. S17). Diffusion distance was proposed as a measure of cell similarity in the original paper [13], and was the most effective of the embeddings used in this study. We see the same trends as in gene expression space; the divergence curve of PROSSTT has positive slope and is consistently above 1, while the Splatter curve has a (clear) negative slope and stays below 1.

After quantifying divergence in both PROSSTT and Splatter simulations, we conclude that the simulations produced by Splatter have inherent characteristics that prevent algorithms that try to find a global structure (like MERLoT and Monocle2) from reaching their full potential. Consequently, we decided to only use simpler topologies for the Splatter benchmark (up to 4 bifurcations), where the impact of short-circuits was less dominant.

Suppl. Note 4 Downstream analysis

Differentially expressed genes detection

After a lineage tree reconstruction has been performed, MERLoT can easily find groups of genes being differentially expressed among different groups of cells. If two groups of cells are provided, e.g cells assigned to two branches in the tree (Fig. 3C)), MERLoT performs a Kruskal-Wallis rank sum test [14] to evaluate which genes in the full expression matrix are differentially expressed on them. If a single subpopulation of cells is provided, the comparison is made against the rest of cells in the data. The entire list of genes is given as output, ordered by the test p-values results. Also, e-values are provided by multiplying the p-values by the number of G genes being tested.

Kruskal-Wallis rank sum test:
Also known as the Kruskal-Wallis one-way analysis of variance, the Kruskal-Wallis rank sum test [14] is a non-parametric method for testing whether the underlying distribution of C samples is identical. The observations are ranked across samples, and a test statistic H is calculated to compare the average rank within each group to the overall average rank. The intuition behind the test is that if samples are identically distributed, then their average ranks will not differ significantly, and the value of the test statistic will be maximized.

Suppl. Note 4.1 GCN reconstruction

MERLoT can be used to study gene-gene expression correlation along every tree branch. In a proof-of-concept we have illustrated how this can be exploited to derive a gene-gene correlation network. In the example showcased in this paper (see Fig. 8 and Figs. S10-S13) genes with similar functions, as characterized by their Gene Ontology annotation, clustered together. Whole clusters were upregulated or downregulated in different parts of the tree, reflecting the multiple differentiation stages and paths available in the process.

Suppl. Note 5 GCNs for Guo 2010

When analyzing datasets containing more than 2 populations of cell types there are more chances to be affected by the Simpson's Paradox, i.e having spurious gene-gene correlations due to an artifact that occurs when taking all cells together in contrast to the correlations that can be recovered when considering subpopulations of specific cell types [15]. The Guo dataset contains a population of progenitors, zygote cells, and 3 mature cell types, TE, PE and EPI cell types. After

reconstructing the lineage tree for this dataset (Fig. 2B) and cells are assigned to the different support nodes in the tree structure we can subdivide them in a trajectory-wise fashion.

Starting from the zygote endpoint we can follow paths to the mature cell types endpoints and define 3 different trajectories: TE, PE and EPI. Following the strategy that was used to analyze the Treutlein dataset, in Figs. S20A, B, and C we show the GCN that can be recovered by using all cells together coloring the genes that are differentially expressed in the terminal branches of each trajectory. Significantly upregulated genes are coloured in shades of red, downregulated genes are coloured in shades of blue and non-differentially expressed genes are coloured in black. Genes that are upregulated in the PE (Figs. S20B) and EPI (Figs. S20C) branches are located in the same region of the GCN with many of the genes being shared between the two trajectories. These two populations emerge from the ICM branch and split away in their last branches. As it was shown in Fig. 3 some genes are upregulated in the EPI branch and downregulated in the PE branch or vice versa. If all cells are taken at the same time to calculate the correlation between pairs of genes, the obtained values can be misleading due to the averaging between the two trajectories.

In Figs. S20D, E and F we show the GCNs that are recovered by using only those cells that lie in the trajectory that goes from the zygote subpopulation of cells to each of the mature cell types. By doing this, we overcome the Simpson's paradox and recover more accurate correlation values for a given trajectory. In the GCNs using all cells, genes like Dab2 are close to the cluster of highly expressed genes regardless of it being upregulated in the PE branch and neutral in the EPI branch. In contrast, we observe that Dab2 appears as a hub node close to the cluster of highly expressed genes in the PE trajectory GCN, while it locates itself close to other neutral genes in the EPI GCN. The more branches a lineage tree contains the more important it becomes to correctly separate multiple trajectories for downstream analysis like GCN or GRN reconstruction.

Suppl. Note 6 MERLoT in $\mathbb{R}^D, D > 3$

In the original Monocle2 paper [16], the authors show in Supplementary Figure 16 their analysis of the haematopoiesis dataset produced by Paul *et al.* [3]. They use 10 components of the DDRTree projection to recover a topology with 5 branching points. We followed this analysis and obtained the DDRTree coordinates. We fitted a scaffold tree and an elastic tree ("auto" mode, with local averaging to 800 cells, and minimum sensitive branch length $\sqrt{N}/2$) on these coordinates and visualized the resulting tree using as annotation the cell types Paul *et al.* assigned to the various clusters.

Monocle2 does a good job of separating the multiple cell fates. On the top of the tree (Fig. S21A) the first branch is composed of multipotent progenitors (in cyan), mixed with more mature cells. This branch separates into the erythroid lineage (purple), the megacaryocyte branch (blue) and the granulocyte/monocyte progenitor (brown) branch, which gives rise to the (mostly) basophil branch as well as three other mixed branches: one with a mixture of dendritic cells (red) and monocytes (orange), one that contains cells from different groups, and one predominantly neutrophilic (magenta)/monocytic branch.

MERLoT however improves quite a bit on this (Fig. S21C and D). In particular, MERLoT separates the megakaryocyte and the dendritic cell lineages much more clearly, despite them being underrepresented (S21C). The erythrocyte branch reflects the Monocle2 assignment, with most cells further away from the tip, while the granulocyte/monocyte internal branch has multiple basophil cells, much like Monocle2. An interesting detail is that while MERLoT also mixes multiple mature cell types with the multipotent progenitors, it does a better job of separating them, even suggesting another sparsely populated branch for mostly neutrophil cells. Finally, MERLoT proposes a split for the common monocyte/neutrophil lineage.

The analysis and plotting can be found in `https://github.com/soedinglab/merlot-scripts`.

Data availability

The 10 simulation sets with 100 simulated differentiations each are available at `http://wwwuser.gwdg.de/~{}compbiol/merlot/`. The code necessary to run the benchmark on the simulations as well as instructions about how to set up a similar benchmark are available at `https://github.com/soedinglab/merlot-scripts`. Formatted expression data for the three datasets in Fig. 2 are available at: `https://github.com/soedinglab/merlot/tree/master/inst/example/`.

Competing interests

The authors declare that they have no competing interests.

Supplementary Figures

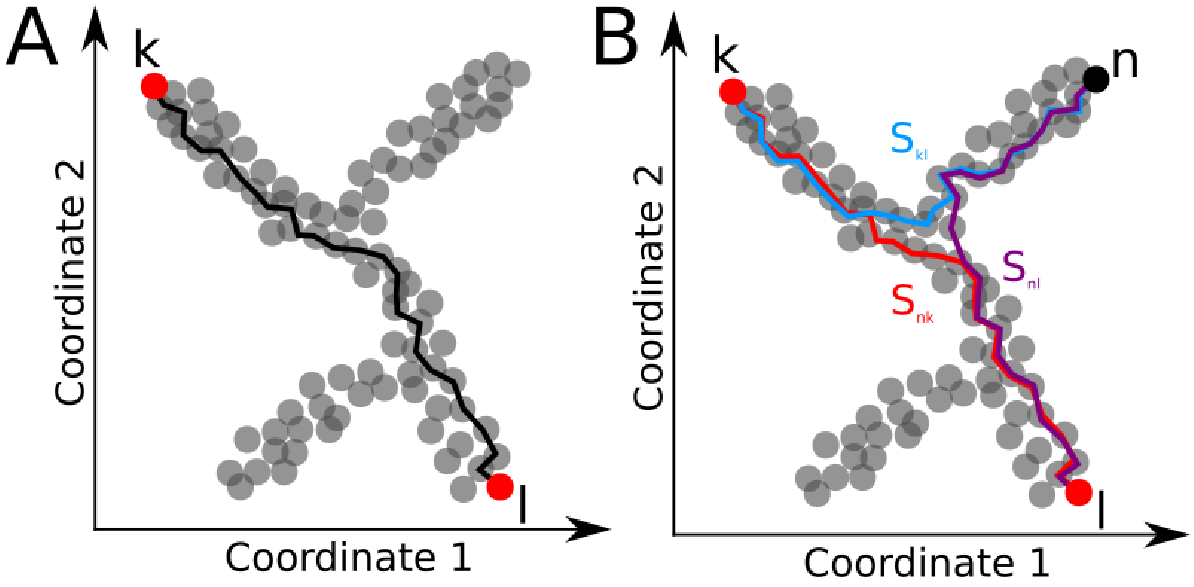

Figure S1: MERLoT's method for finding endpoints: (A) Pair of cells that maximize the length of the shortest path distances matrix in the dataset. **(B)** Every cell n, not in the shortest path between already found endpoints is evaluated according to: $s_{\mathcal{E}}(n) := 0.5 \times \min\{S_{kn} + S_{nl} - S_{kl}\ :\ k, l \in \mathcal{E}\}$. S_ij represents the number of nodes in the shortest path between i, j. The new endpoint to be added to the tree structure is the one that maximizes its $s_{\mathcal{E}}(n)$ value respect to the other cells in the dataset.

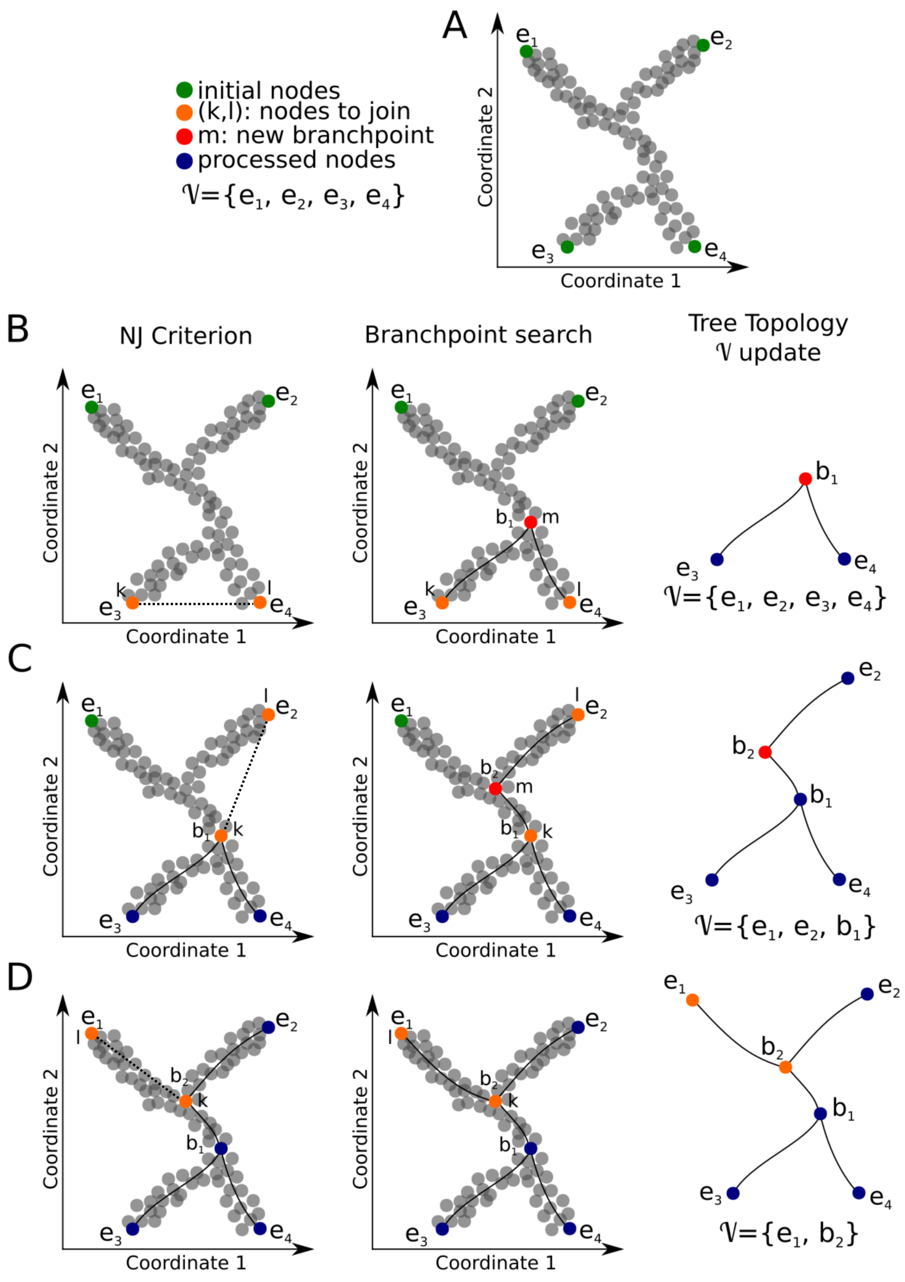

Figure S2: MERLoT's method for finding branchpoints and tree connectivity: The algorithm has two steps (1) Neighbour Joining (NJ) Criterion and (2) branchpoint search. The NJ criterion is applied over the $\mathcal{V}$ vector which is initially set to the set of endpoints (green). In every NJ iteration, two k, l nodes are selected to be joined (dashed lines) for which a branchpoint m is found (red) and the edges between the branchpoint and the joined nodes are added to the topology. The $\mathcal{V}$ vector is updated in every iteration by subtracting the k and l nodes and adding the m node. The procedure stops when $|\mathcal{V}|$=2. **(A)** $\mathcal{V}$ is initialized with the endpoints e1-e4. **(B)** First iteration: e3 and e4 and joined through b1. e3-b1 and e4-b1 edges are added to the tree. e3 and e4 are deleted from $\mathcal{V}$ and b1 is added instead. **(C)** Second iteration: e2 and b1 and joined through b2. e2-b2 and b1-b2 edges are added to the tree. e2 and b1 are deleted from $\mathcal{V}$ and b2 is added instead. **(D)** Third iteration: $|\mathcal{V}|$=2, procedure stops and the b2-e1 edge is added to the tree.

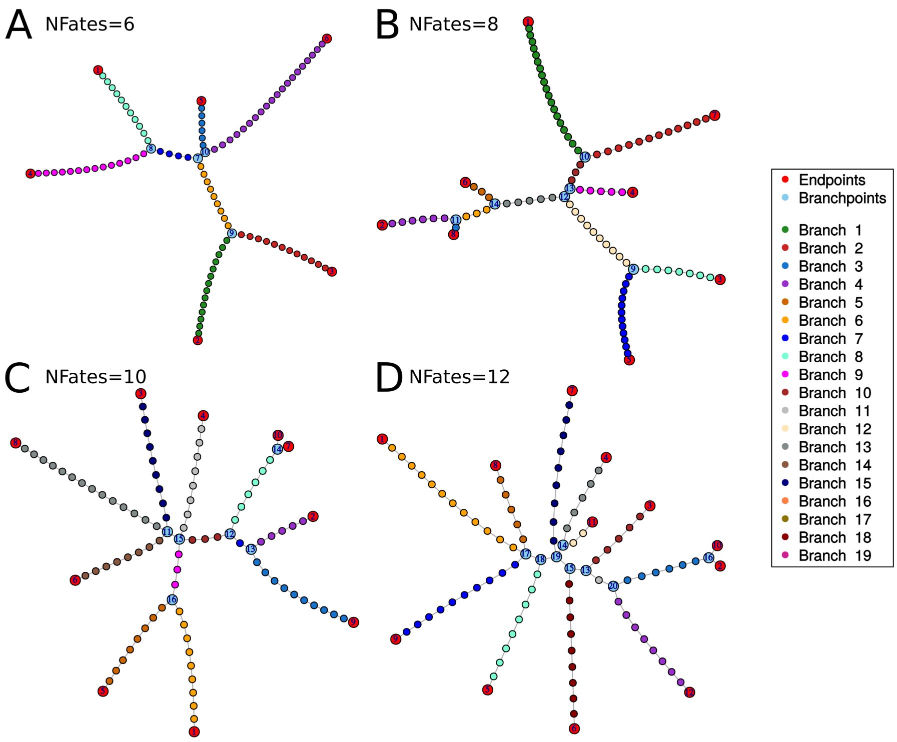

Figure S3: Topology plots: In cases where the dimensionality reduction happens in more than three dimensions, MERLoT can plot a schematic representation of the lineage tree.

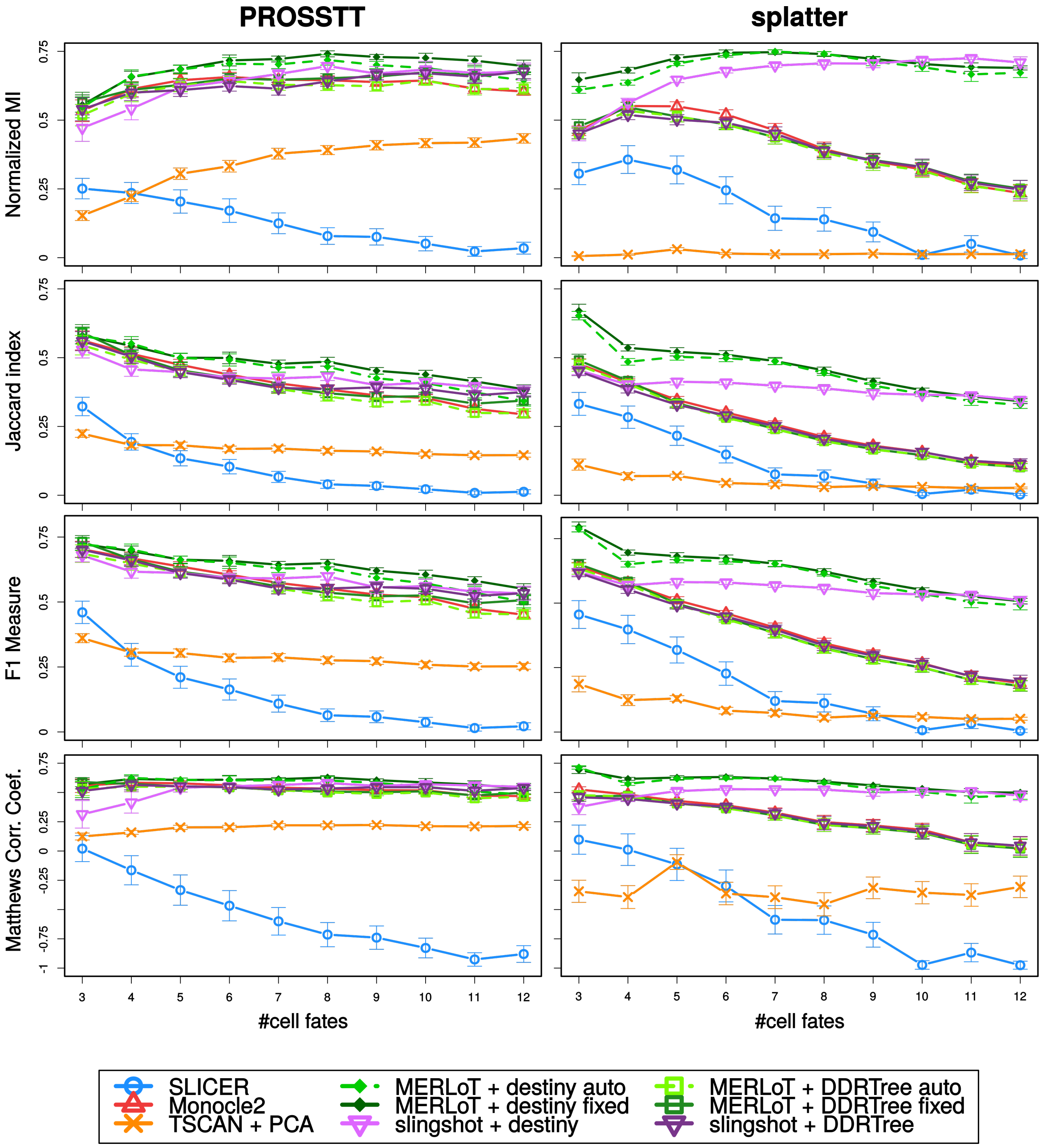

Figure S4: Branch assignment benchmarking. We calculated different scores to assess how good the different methods are at assigning cells to the different measures on the PROSSTT "lean" (left) and Splatter (right) simulation sets. From top to bottom, NMI, Jaccard Index, F1 measure, and MCC. The error bars are 95% confidence intervals assuming the prediction scores are normally distributed.

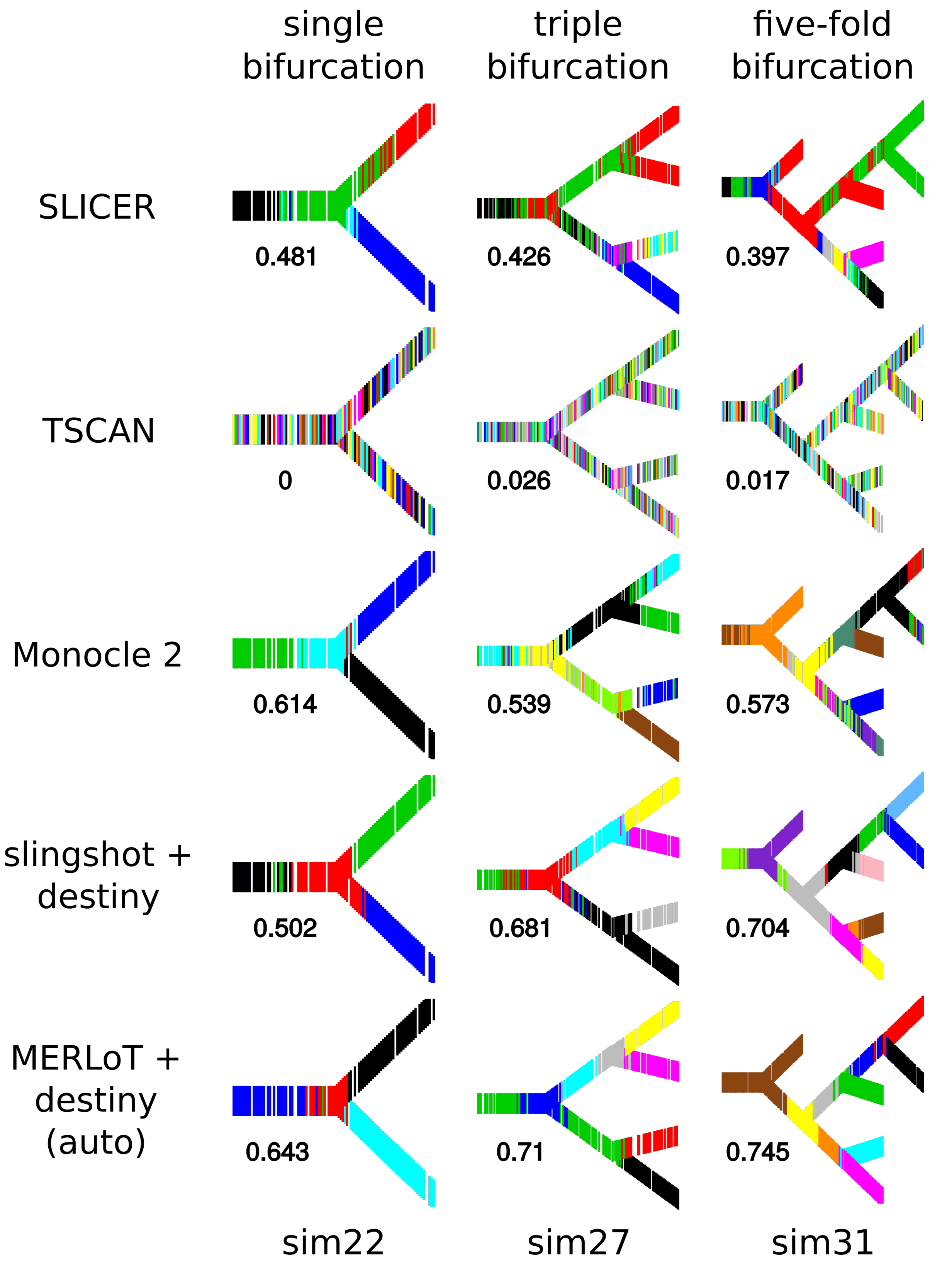

Figure S5: Examples of branch assignments for MERLoT, Monocle2, and SLICER. We show three typical examples from the single (left), triple (middle) and five-fold (right) bifurcation benchmark sets (simulation names at bottom). In each panel we show the lineage tree of the simulated tree, with vertical lines representing the cells, ordered by pseudotime. The color of each line is the branch label predicted for it by the respective method. The NMI of each prediction is in the top left of each panel. We picked examples with NMI values near the average values for each tool and tree topology.

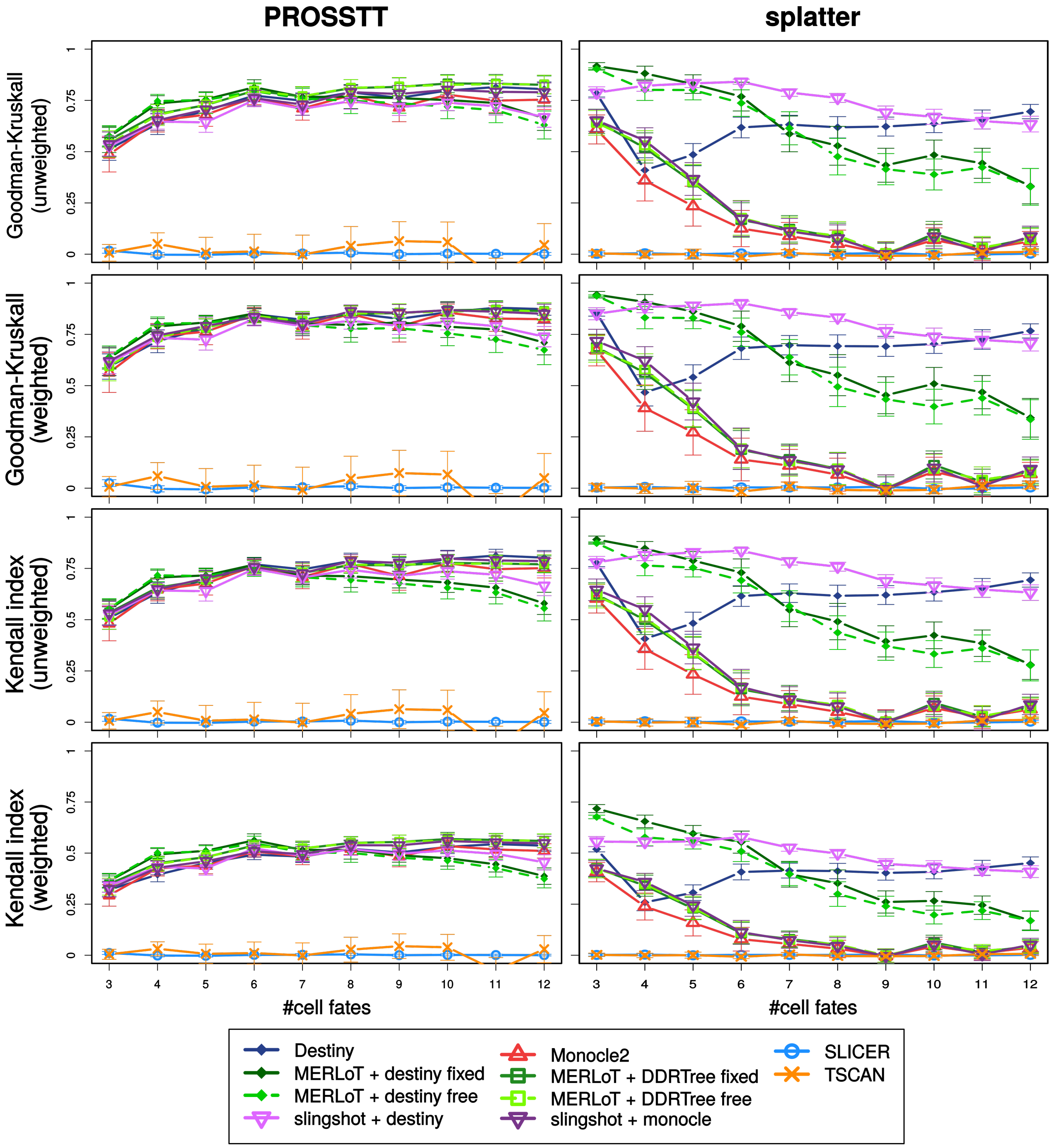

Figure S6: Pseudotime assignment benchmarking. We calculated different scores to assess how good the different methods are at assigning pseudotime values to cells along the longest sub tree in the PROSSTT "lean" (left) and Splatter (right) simulation sets. From top to bottom, Goodman-Kruskal index (unweighted and weighted), and Kendall index (unweighted and weighted). The error bars are 95% confidence intervals assuming the prediction scores are normally distributed.

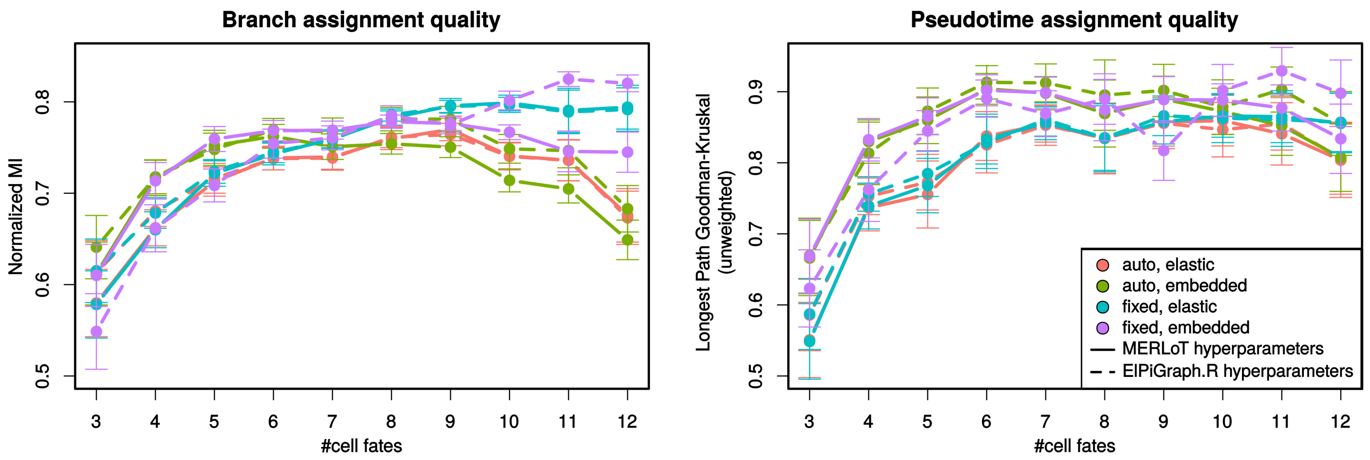

Figure S7: Comparison of hyperparameter performance: We compared the average performance of different MERLoT flavours in vanilla mode against MERLoT with ElPiGraph.R elasticity hyperparameters. The comparison is on the "deep" benchmark. The different colours are different MERLoT flavours; solid lines denote MERLoT with MERLoT hyperparameters; dashed lines denote MERLoT with ElPiGraph.R hyperparameters. The error bars are 95% confidence intervals assuming the prediction scores are normally distributed. All MERLoT flavours were ran on local averaging mode, as was the whole benchmark.

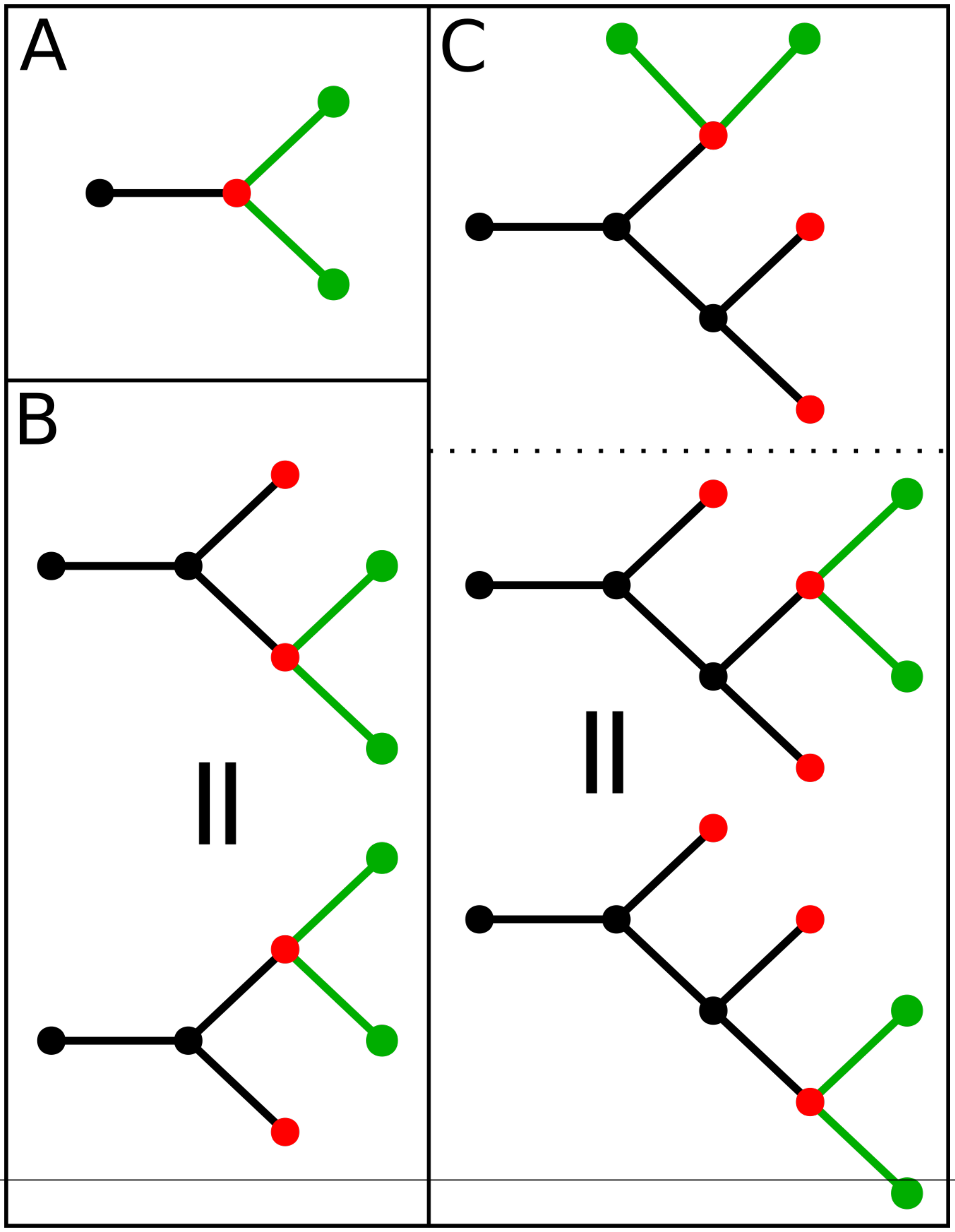

Figure S8: Topology Complexity. **(A)** With one bifurcation only one topology is possible. **(B)** With two bifurcations two topologies are possible. However, they are equivalent in terms of length of the longest subtree. **(C)** More topologies are possible. For the upper case in panel B, three more topologies can be constructed by adding a new bifurcation. Not all topologies are equivalent in terms of the length of the longest sub tree.

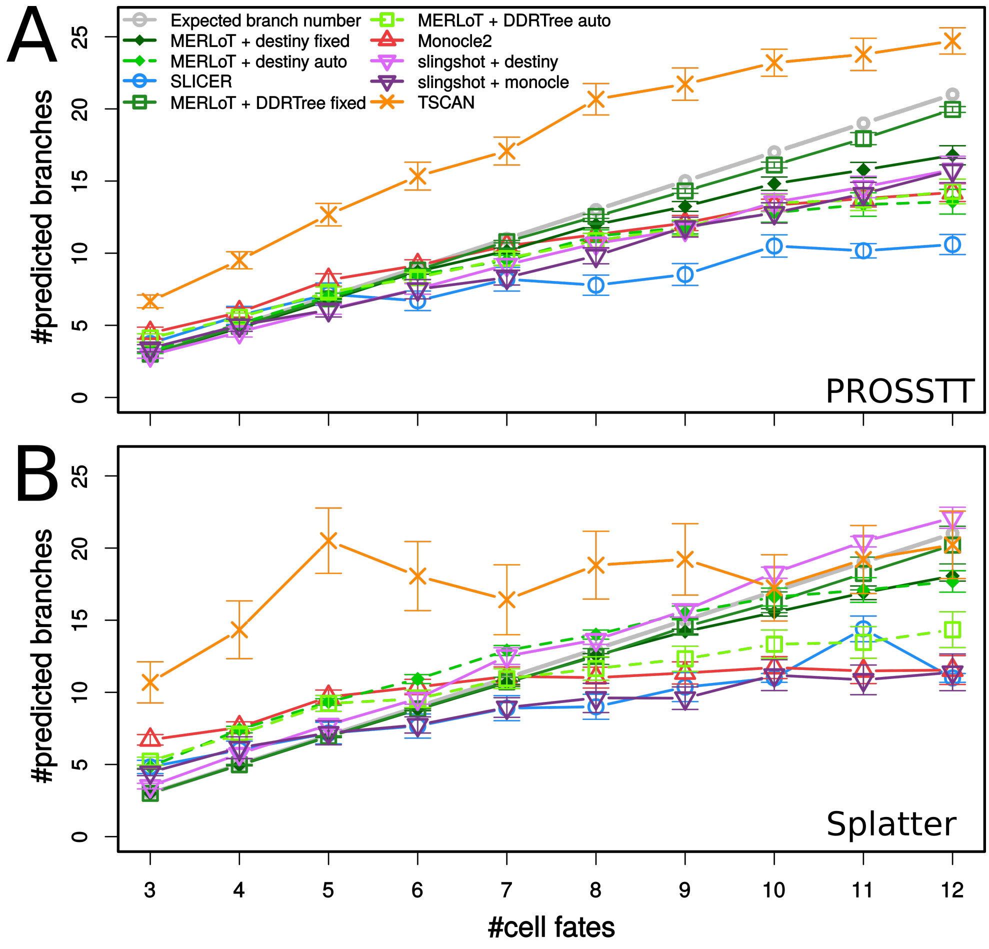

Figure S9: Number of predicted branches in simulated datasets. We compare how many branches out of the true number of generated branches each tool is able to predict. The true simulated number of branches y shown in gray, and the different tools are shown in different colors and symbols as described in the legend. **(A)** PROSSTT “lean” set. **(B)** Splatter set. The error bars are 95% confidence intervals assuming the prediction scores are normally distributed.

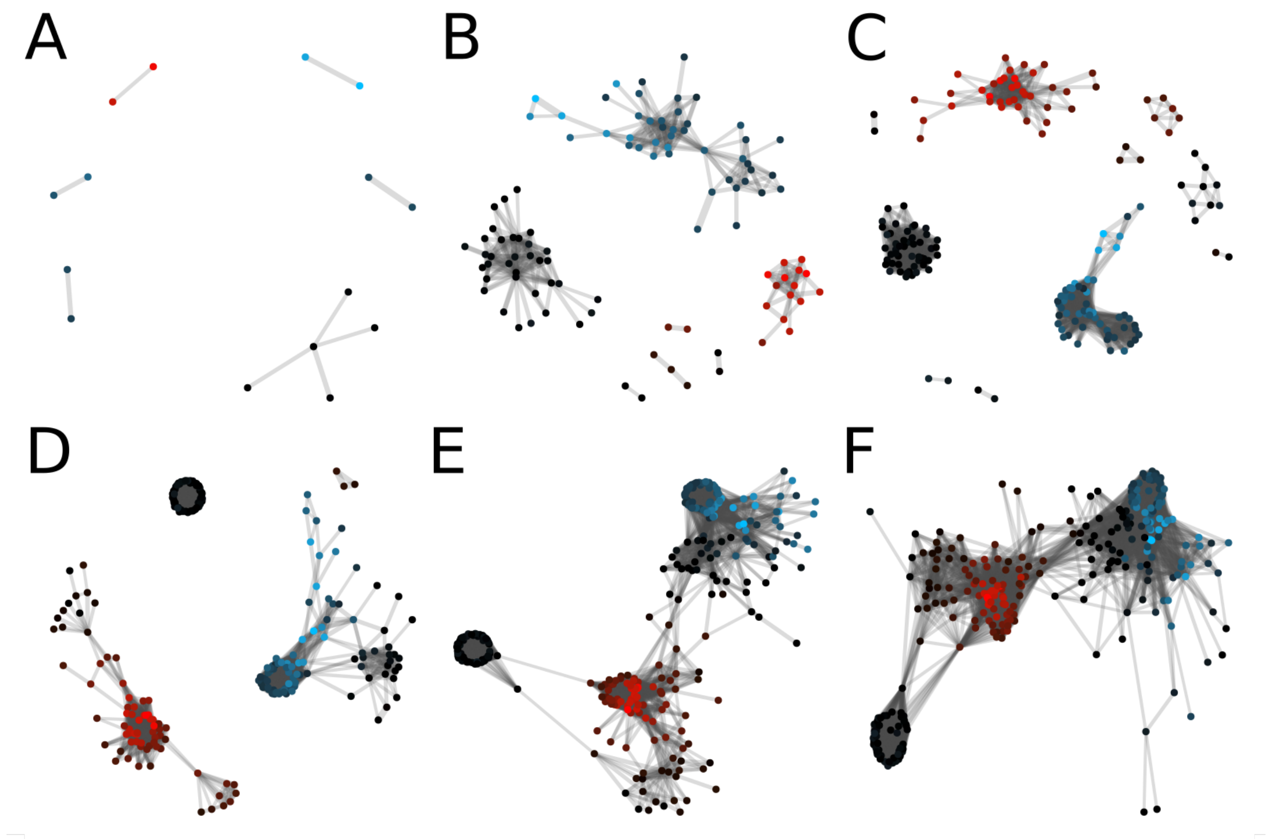

Figure S10: Fibroblasts differentially expressed genes mapped in the GCN structure. GCN is reconstructed using different thresholds for Pearson's correlation coefficient between genes: **(A)** 0.9, **(B)** 0.8, **(C)** 0.7, **(D)** 0.6, **(E)** 0.5, **(F)** 0.4. Genes in red are significantly upregulated. Genes in blue are significantly downregulated. Genes in black are not significantly differentially expressed.

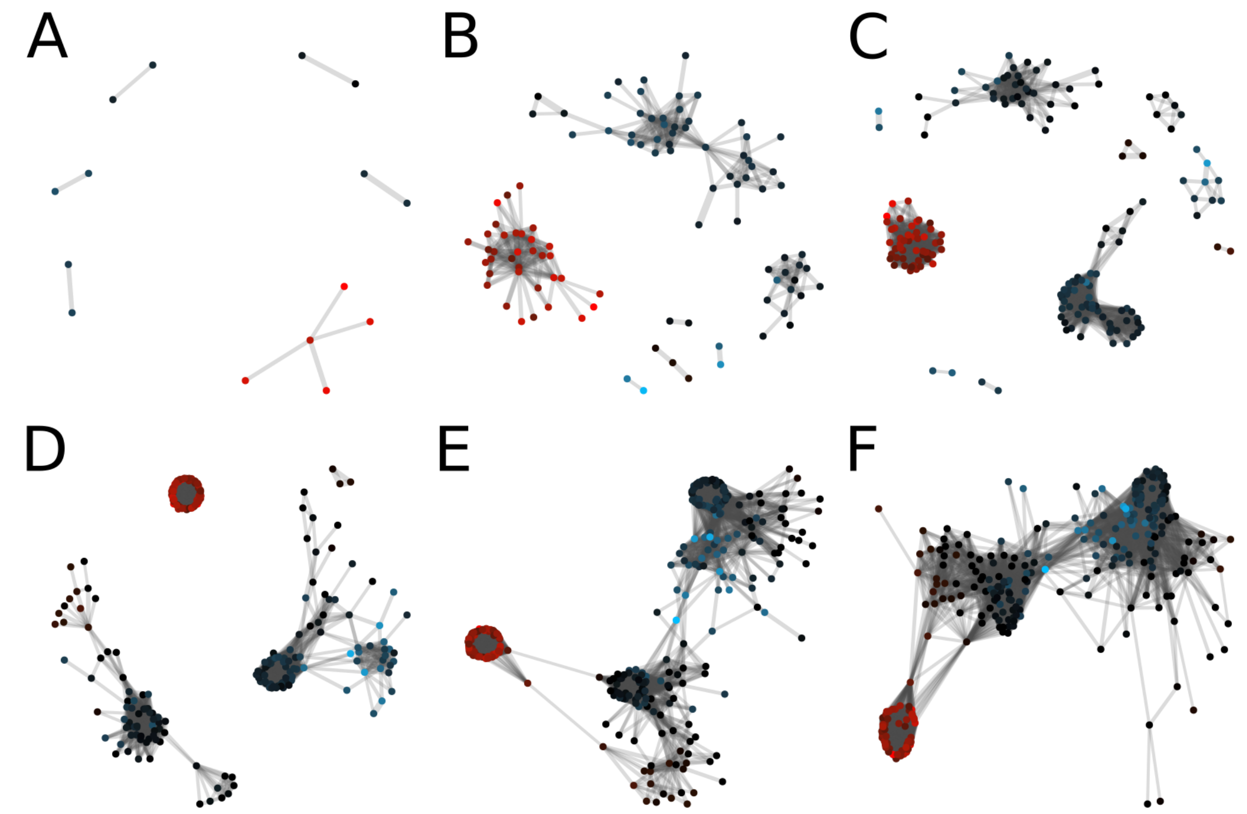

Figure S11: Neurons differentially expressed genes mapped in the GCN structure. GCN is reconstructed using different thresholds for Pearson's correlation coefficient between genes: **(A)** 0.9, **(B)** 0.8, **(C)** 0.7, **(D)** 0.6, **(E)** 0.5, **(F)** 0.4. Genes in red are significantly upregulated. Genes in blue are significantly downregulated. Genes in black are not significantly differentially expressed.

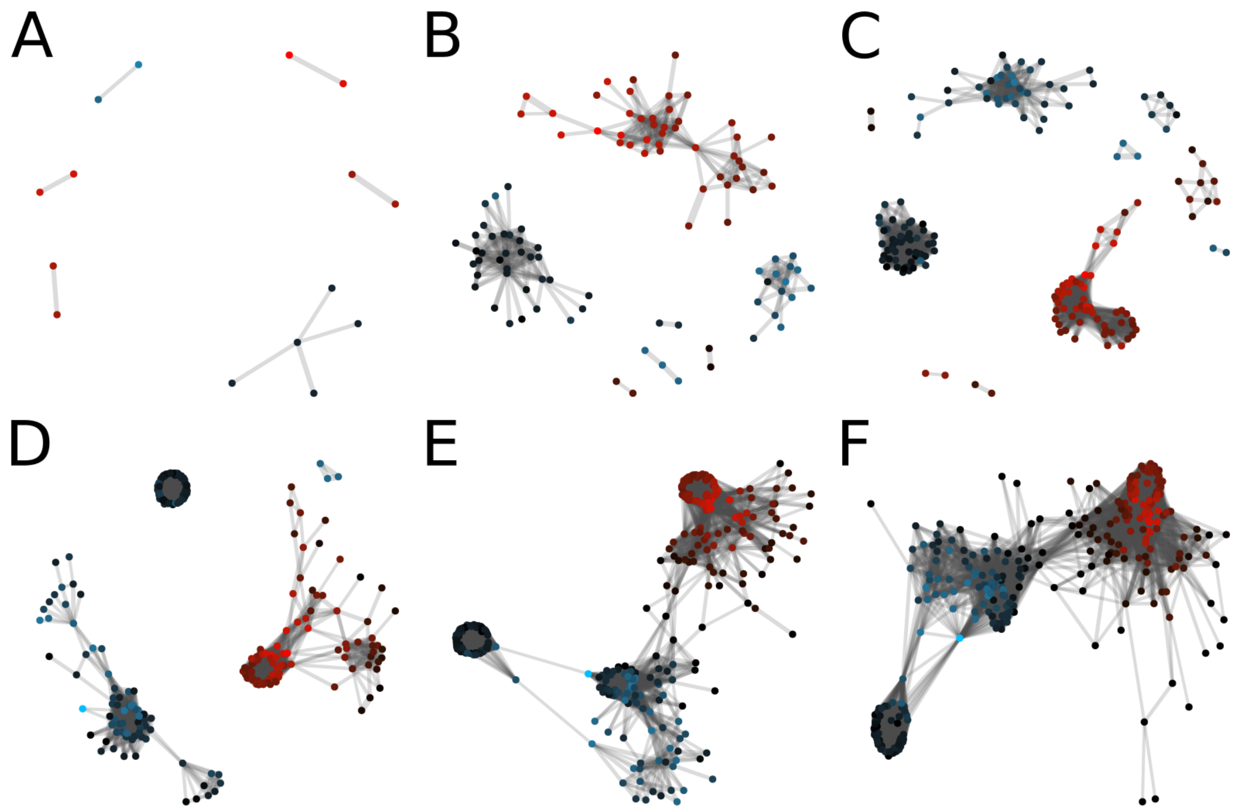

Figure S12: Myocytes differentially expressed genes mapped in the GCN structure. GCN is reconstructed using different thresholds for Pearson's correlation coefficient between genes: **(A)** 0.9, **(B)** 0.8, **(C)** 0.7, **(D)** 0.6, **(E)** 0.5, **(F)** 0.4. Genes in red are significantly upregulated. Genes in blue are significantly downregulated. Genes in black are not significantly differentially expressed.

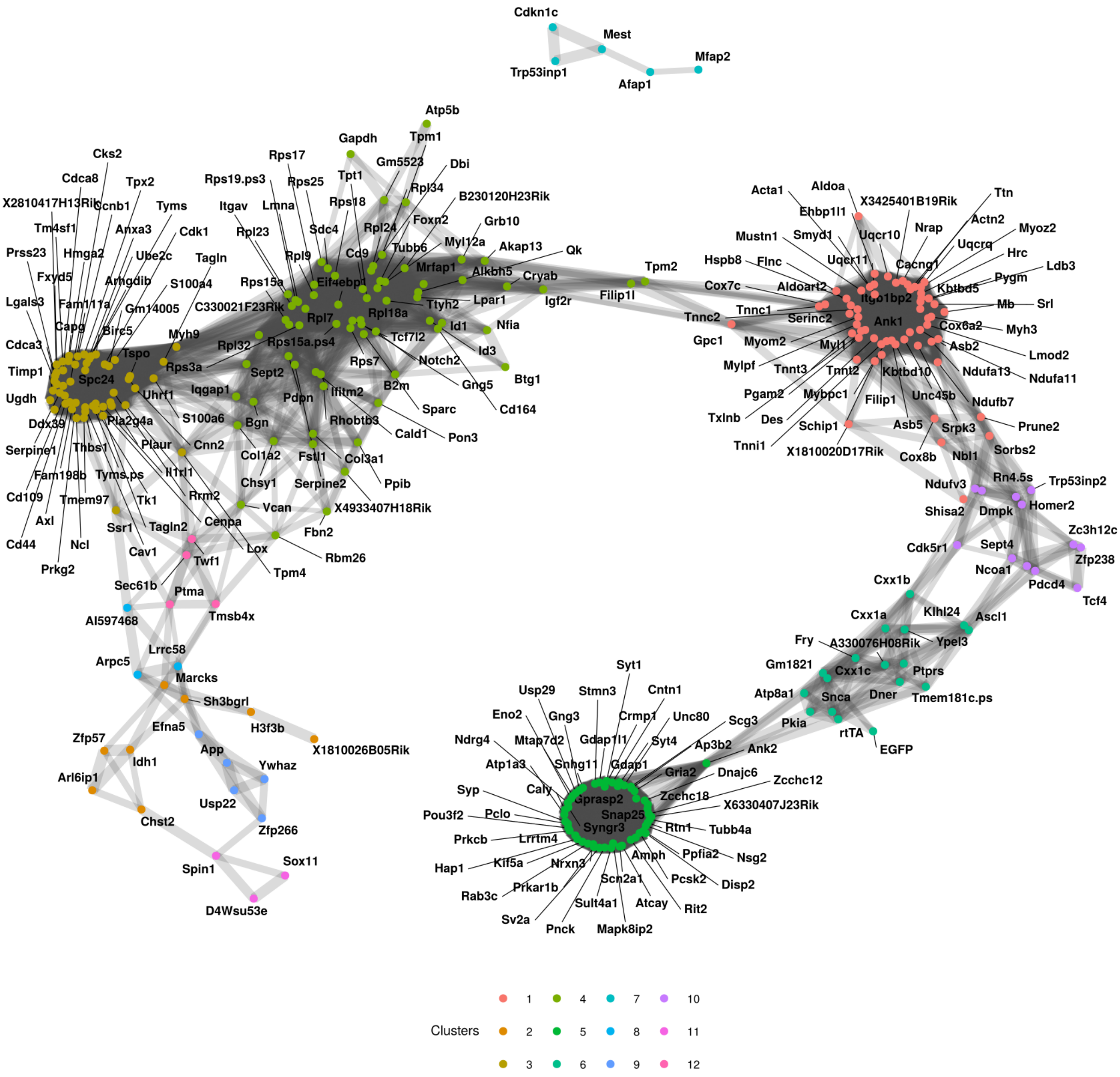

Figure S13: Labeled GCN. Genes were clustered using the "walktrap" algorithm. Groups of genes are coloured according to the clusters found in the network structure.

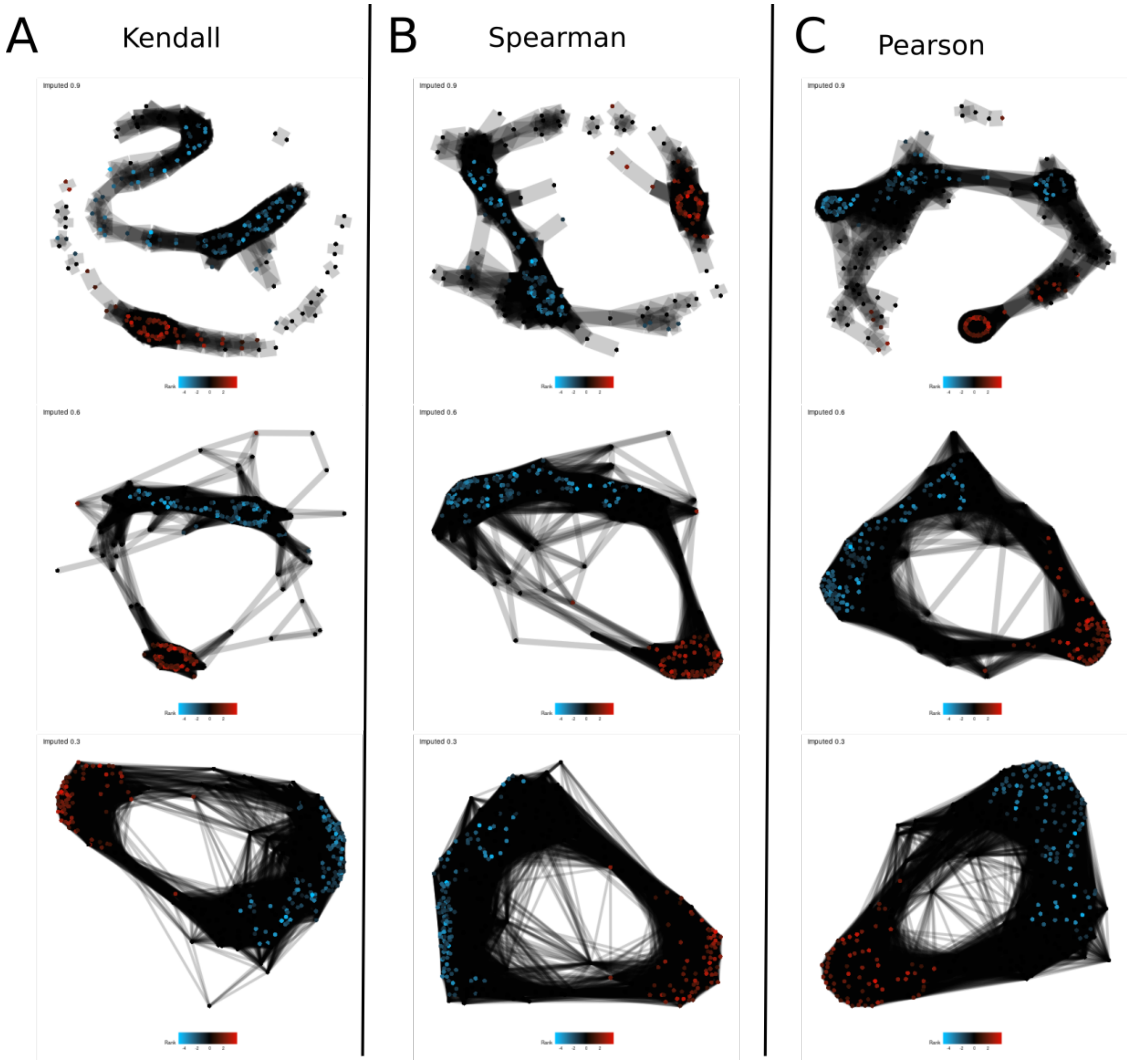

Figure S14: GCN - other measures. Reconstruction of the Treutlein GCN with different correlation measures. **(A)** Kendall's τ coefficient, **(B)** Spearman's ρ, **(C)** Pearson's r. From top to bottom, each row has a different cut-off for the inclusion of edges. Top row is 0.9, middle is 0.6 and bottom is 0.3. The top row of column C is the configuration presented in the main paper. Repeating the GCN reconstruction with different correlation measures produces very similar results, albeit without the clear clustering suggested by the Pearson correlation coefficient analysis. While a threshold of 0.9 was appropriate for the Pearson correlation, it seems as if lower cut-offs might be required for the Spearman and Kendall coefficients.

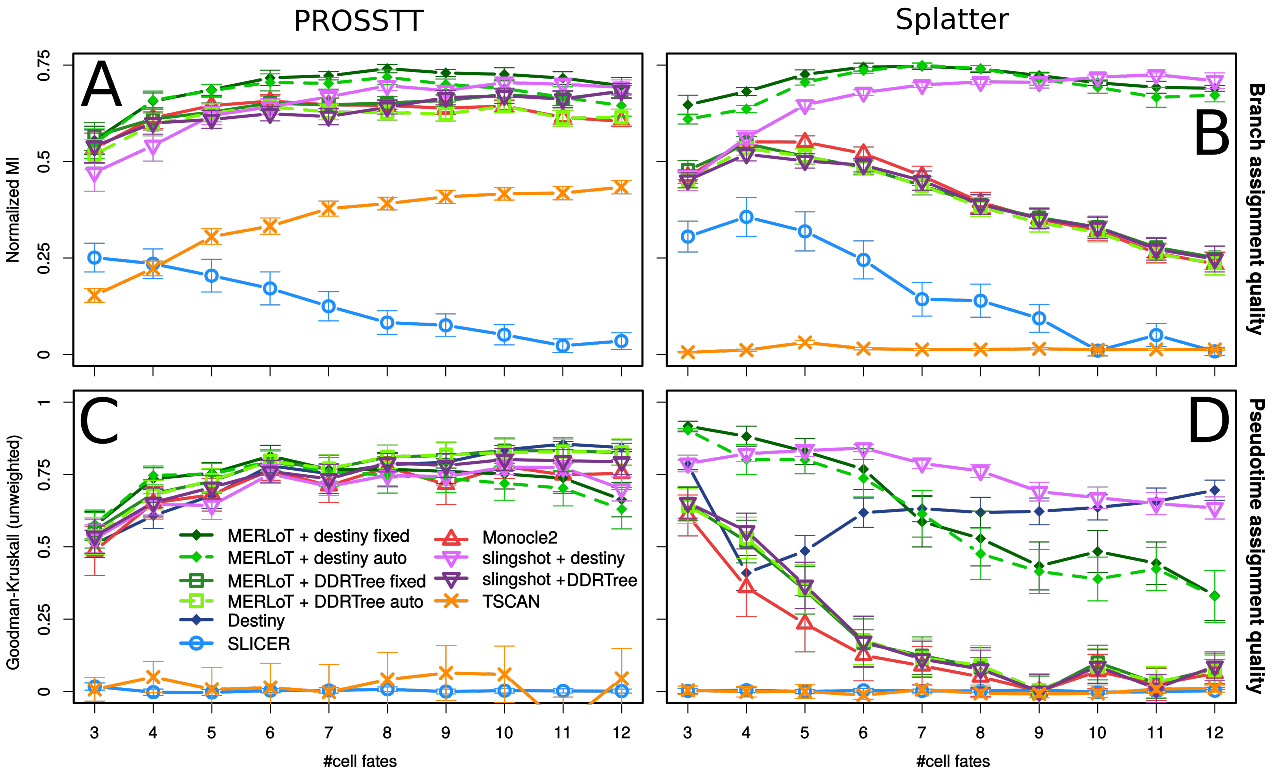

Figure S15: Benchmark results. (A)-(D) Branch assignment (upper panels) and pseudotime assignment (lower panels) comparison using Monocle2, SLICER, TSCAN, Slingshot, and MERLoT using both PROSSTT (left) and Splatter (right) simulations. Slingshot and MERLoT are used in combination with DDRTree and diffusion map (Destiny) coordinates. Compare with Fig. 5. The error bars are 95% confidence intervals assuming the prediction scores are normally distributed.

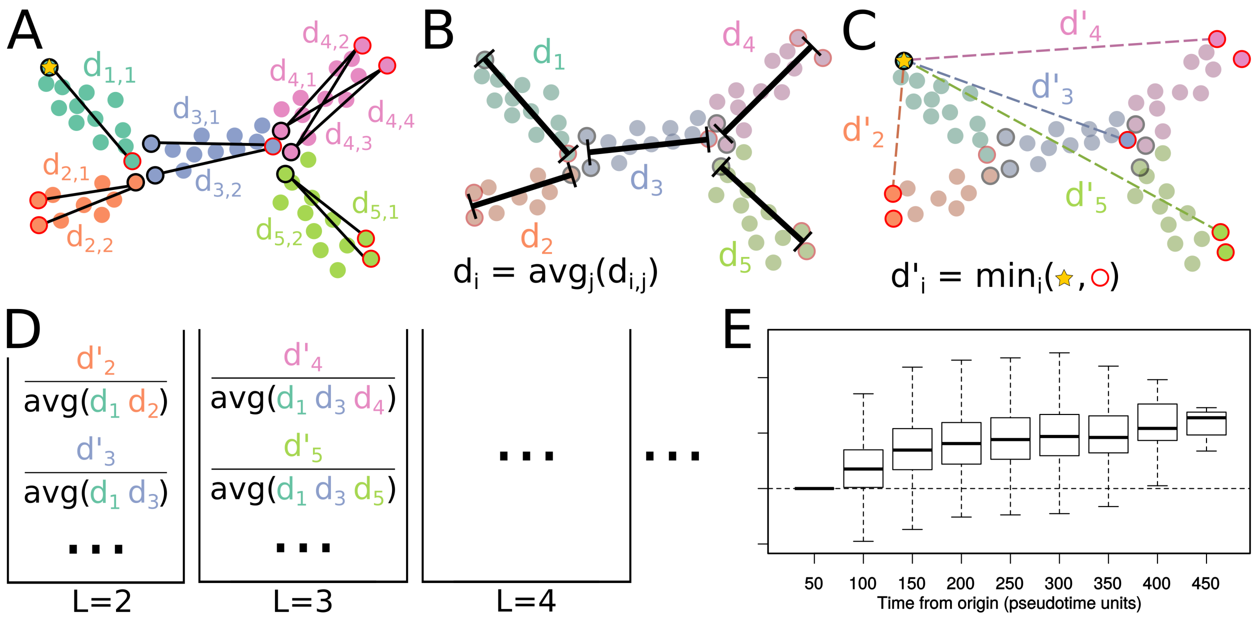

Figure S16: Divergence analysis. (A) We locate the cells with minimum and maximum pseudotime in each branch and calculate their pairwise distances. **(B)** The average of these distances is the branch length. **(C)** We calculate the minimum direct distance of the origin of the differentiation (minimum pseudotime of first branch) to the other waypoints (maximum pseudotime of each branch). **(D)** We take all on-tree paths from the origin to a waypoint and normalize the minimum direct distance via the average branch length in the path. After repeating steps (A)-(C) for all simulations in the set, we group these values by path length (in branches). **(E)** The boxplots of each path bin constitute the divergence curve of the simulations. If the branches all have the same pseudotime length, then distance to waypoints is equivalent to the pseudotime that has passed since the origin.

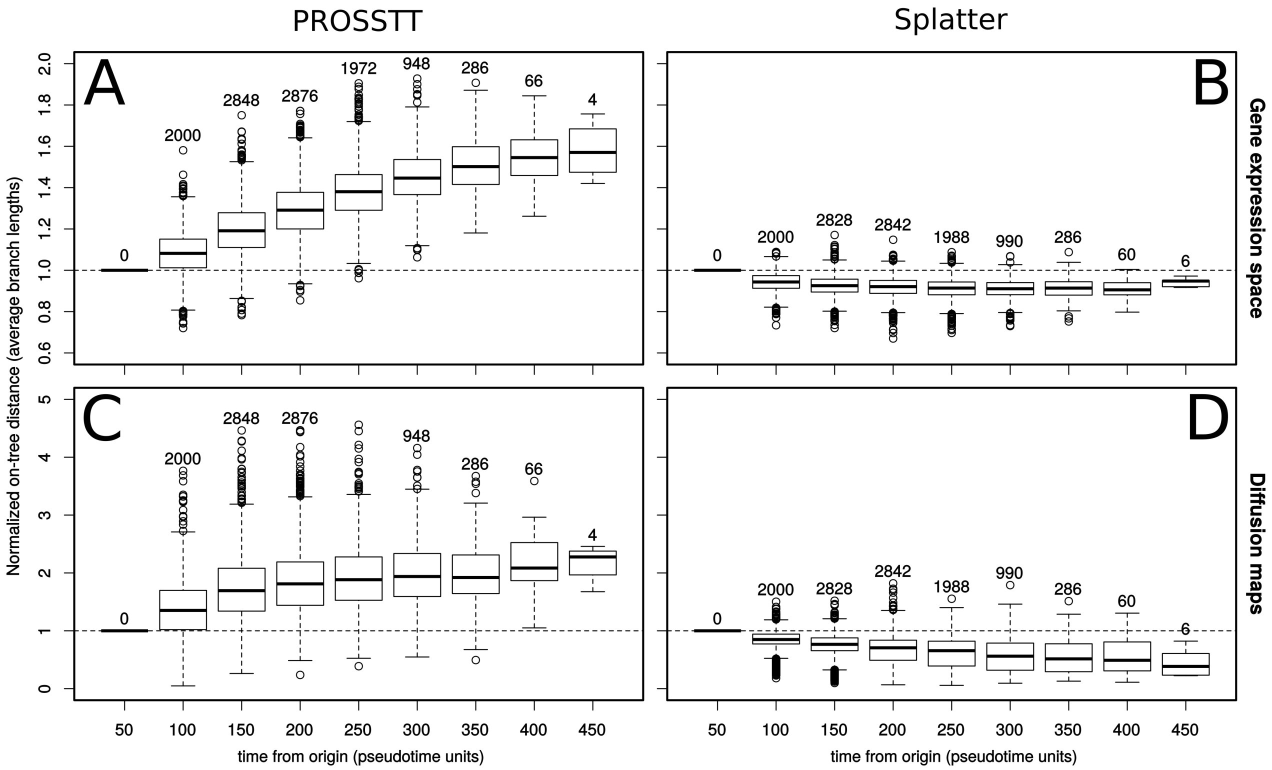

Figure S17: Divergence analysis results. Divergence analysis for PROSSTT (left) and Splatter (right) simulations in gene expression space (upper panels) or in diffusion space (lower panels).

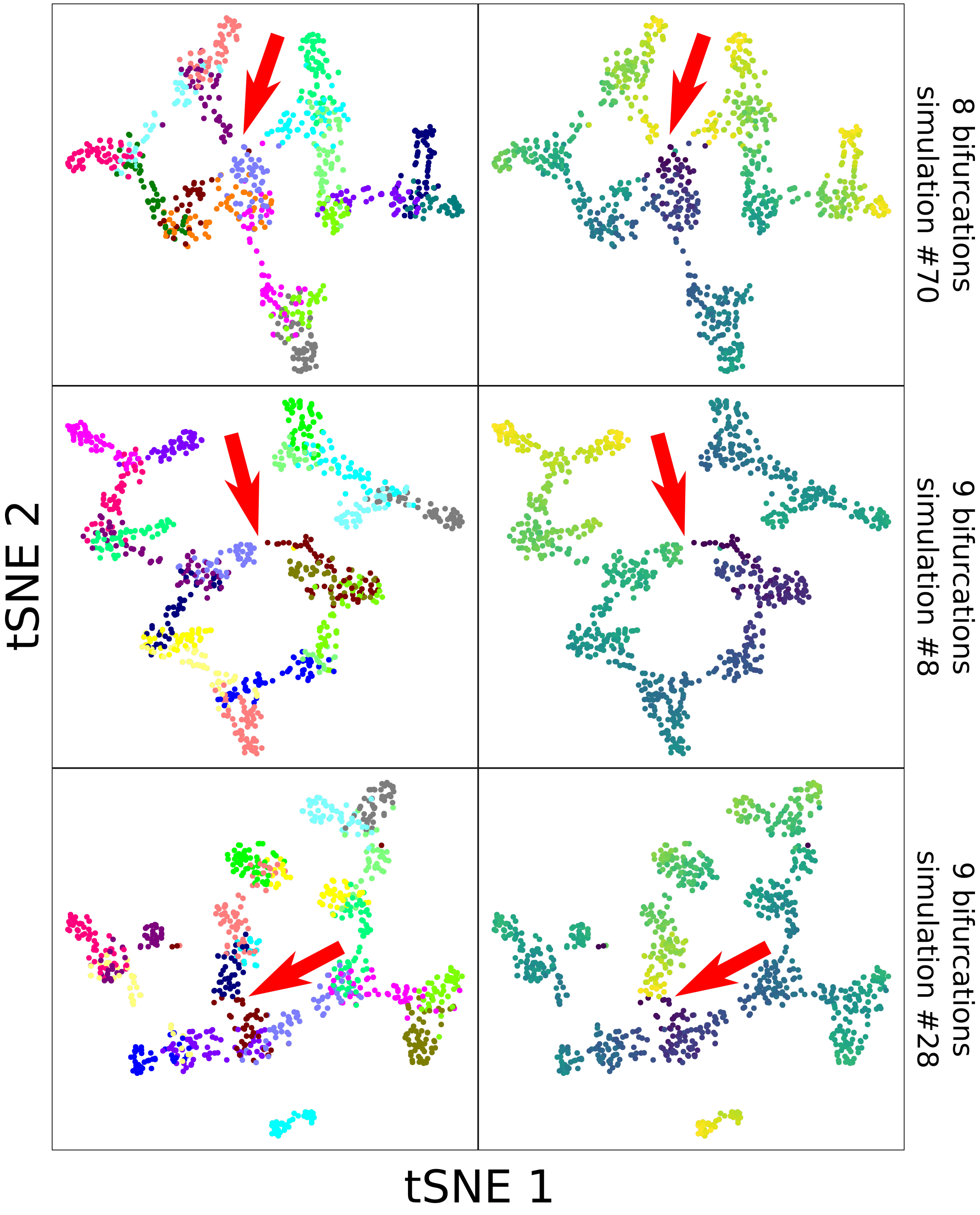

Figure S18: Short circuits. tSNE plots of Splatter simulations that show short-circuits. On the left side the colors denote the branches. On the right side the color denotes the pseudotime, from low (dark blue) to high (yellow). The red arrows point to the exact points where the short-circuit takes place. In all cases, cells with low and high pseudotime are so close to each other that the shape of the lineage tree is not clear, leading to problems in the tree reconstruction.

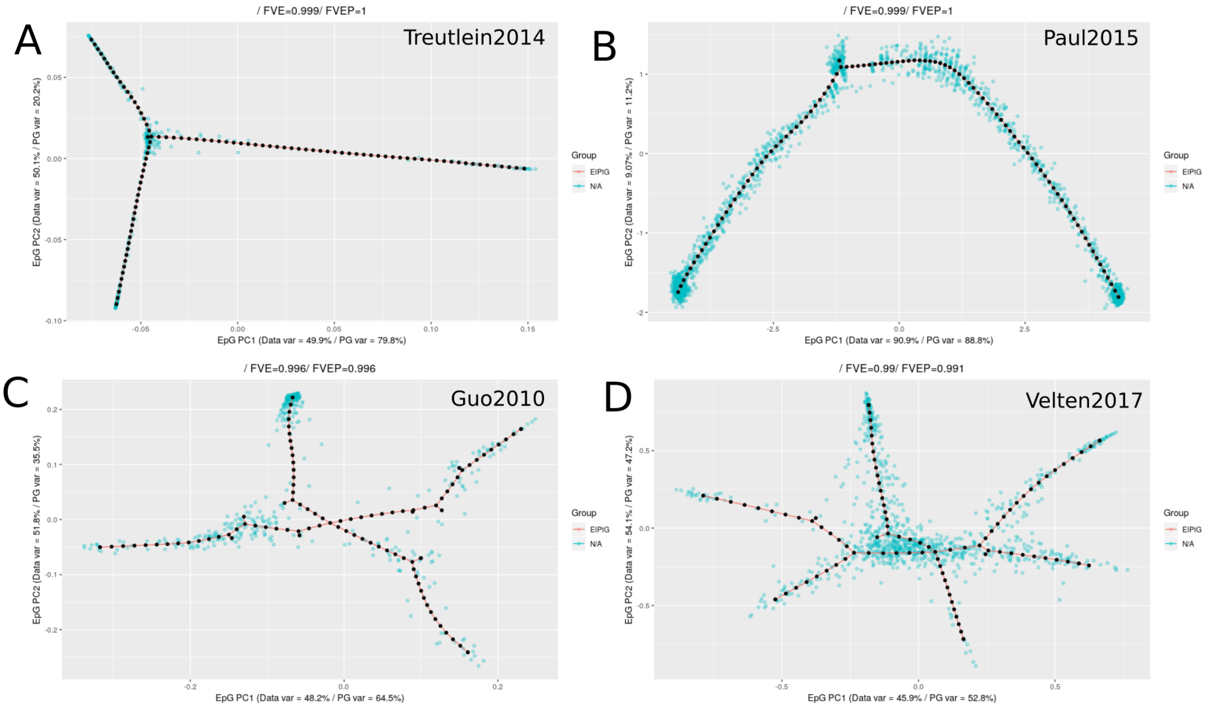

Figure S19: ElPiGraph tree reconstructions. We performed tree reconstructions for the datasets used as examples in the manuscript using the computeElasticPrincipalTree function from ElPiGraph using default parameters. **(A)** Treutlein dataset as used in Fig 5A using diffusion coordinates calculated by the Destiny package. **(B)** Paul dataset as used in Fig. 2D using DDR3 coordinates calculated by the Monocle2 package. **(C)** Guo dataset as used in Fig. 2E using diffusion coordinates calculated by the Destiny package. **(D)** Velten dataset as used in Fig. 2F using coordinates calculated by the STEMNET package.

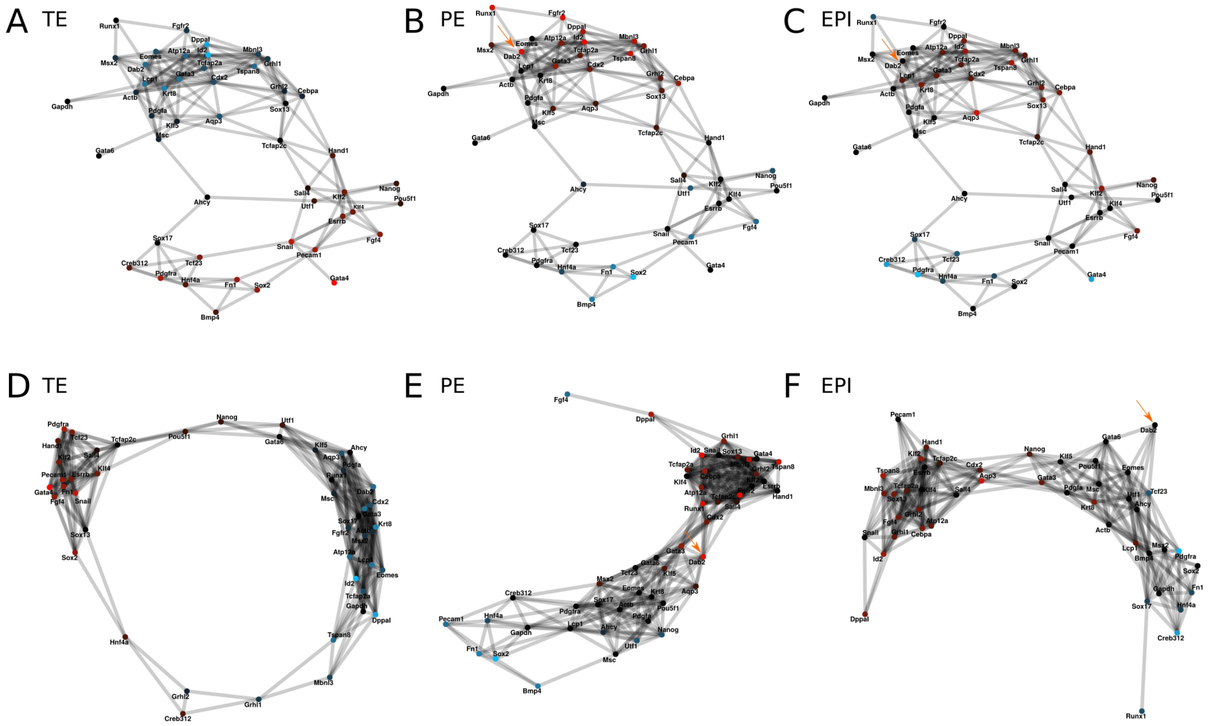

Figure S20: Zygote to Blastocyst GCNs (Guo 2010). We performed GCNs reconstructions using all cells such that we show differentially upregulated genes in shades of red and downregulated genes in shades of blue for **(A)** TE branch, **(B)** PE branch, **(C)** EPI branch. The same color schema was used for GCNs using trajectory specific cells for **(D)** TE trajectory, **(E)** PE trajectory, **(F)** EPI trajectory.

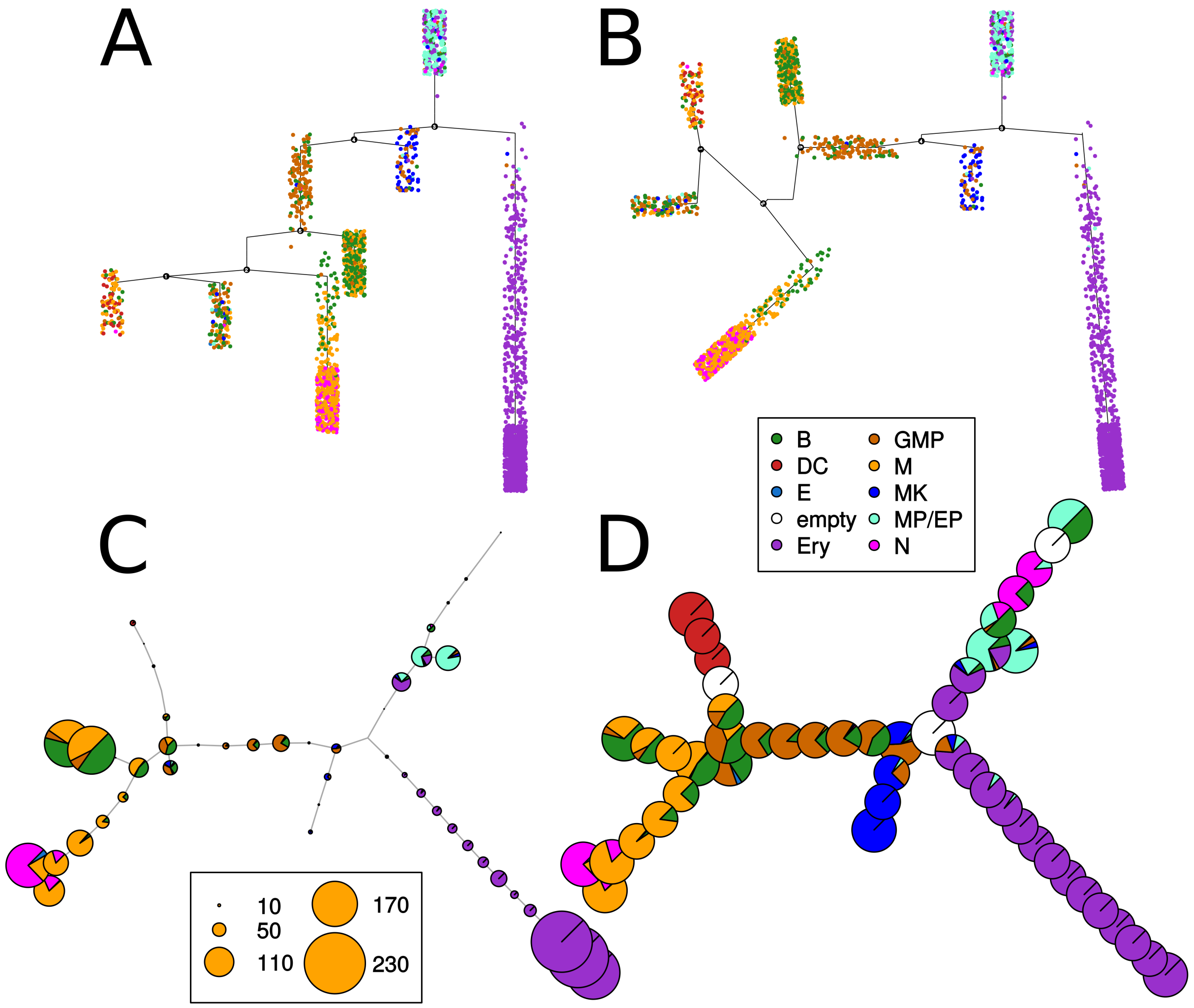

Figure S21: MERLoT improves upon Monocle2's reconstruction. (A) The flattened tree schematic provided by Monocle2. The erythrocyte branch (purple) is clearly separated, and while other branches contain similar progenitor groups, the separation is not optimal. **Abbreviations:** B: basophil (green); DC: dendritic cell (red); E: eosinophil (light blue); Ery: erythrocyte (purple); GMP: granulocyte and monocyte progenitor (brown); M: monocyte; MK: megakaryocyte; MP/EP: multipotent myeloid and erythroid progenitors; N: neutrophil (orange) **(B)** The same topology but arranged in slightly different manner to facilitate comparison with the MERLoT reconstructions. **(C)** The MERLoT reconstruction of the same tree (based on the same coordinates): The branches are more homogeneous. The colour composition of each pie chart corresponds to the types of cells assigned to that elastic tree node. The size of the pie charts is proportional to the number of cells assigned to each node. **(D)** The same reconstruction but without the number of cells.

References

[1] Saitou, N. and Nei, M. (1987) The neighbor-joining method: a new method for reconstructing phylogenetic trees.. *Molecular biology and evolution,* **4**(4), 406–425.

[2] Guo, G., Huss, M., Tong, G. Q., Wang, C., Sun, L. L., Clarke, N. D., and Robson, P. (2010) Resolution of cell fate decisions revealed by single-cell gene expression analysis from zygote to blastocyst. *Developmental cell,* **18**(4), 675–685.

[3] Paul, F., Arkin, Y., Giladi, A., Jaitin, D. A., Kenigsberg, E., Keren-Shaul, H., Winter, D., Lara-Astiaso, D., Gury, M., Weiner, A., et al. (2015) Transcriptional heterogeneity and lineage commitment in myeloid progenitors. *Cell,* **163**(7), 1663–1677.

[4] Grün, D., Kester, L., and Van Oudenaarden, A. (2014) Validation of noise models for single-cell transcriptomics. *Nature methods,* **11**(6), 637.

[5] Harris, K. D., Bengtsson Gonzales, C., Hochgerner, H., Skene, N. G., Magno, L., Katona, L., Somogyi, P., Kessaris, N., Linnarsson, S., and Hjerling-Leffler, J. (2017) Classes and continua of hippocampal CA1 inhibitory neurons revealed by single-cell transcriptomics. *bioRxiv,*.

[6] Papadopoulos, N., Parra, R. G., and Soeding, J. (2019) PROSSTT: Probabilistic simulation of single-cell RNA-seq data for complex differentiation processes.. *Bioinformatics,*.

[7] Vinh, N. X., Epps, J., and Bailey, J. (December, 2010) Information Theoretic Measures for Clusterings Comparison: Variants, Properties, Normalization and Correction for Chance. *J. Mach. Learn. Res.,* **11**, 2837–2854.

[8] Campello, R. J. G. B. and Hruschka, E. R. (2009) On comparing two sequences of numbers and its applications to clustering analysis. *Information Sciences,* **179**(8), 1025–1039.

[9] Mao, Q., Wang, L., Goodison, S., and Sun, Y. (2015) Dimensionality Reduction Via Graph Structure Learning. In *Proceedings of the 21th ACM SIGKDD International Conference on Knowledge Discovery and Data Mining* New York, NY, USA: ACM KDD '15 pp. 765–774.

[10] Street, K., Risso, D., Fletcher, R. B., Das, D., Ngai, J., Yosef, N., Purdom, E., and Dudoit, S. (Jun, 2018) Slingshot: cell lineage and pseudotime inference for single-cell transcriptomics. *BMC Genomics,* **19**(1), 477.

[11] Hastie, T. and Stuetzle, W. (1989) Principal Curves. *Journal of the American Statistical Association,* **84**(406), 502–516.

[12] Scrucca, L., Fop, M., Murphy, T. B., and Raftery, A. E. (2016) mclust 5: clustering, classification and density estimation using Gaussian finite mixture models. *The R Journal,* **8**(1), 205–233.

[13] Haghverdi, L., Buettner, F., and Theis, F. J. (2015) Diffusion maps for high-dimensional single-cell analysis of differentiation data. *Bioinformatics,* **31**(18), 2989–2998.

[14] Kruskal, W. H. and Wallis, W. A. (1952) Use of Ranks in One-Criterion Variance Analysis. *Journal of the American Statistical Association,* **47**(260), 583–621.

[15] Trapnell, C. (2015) Defining cell types and states with single-cell genomics. *Genome research,* **25**(10), 1491–1498.

[16] Qiu, X., Mao, Q., Tang, Y., Wang, L., Chawla, R., Pliner, H. A., and Trapnell, C. (2017) Reversed graph embedding resolves complex single-cell trajectories. *Nature methods,* **14**(10), 979–982.
